# Natural lipid nanoparticles extracted from *Morus nigra* L. leaves for targeted treatment of hepatocellular carcinoma *via* the oral route

**DOI:** 10.1186/s12951-023-02286-3

**Published:** 2024-01-03

**Authors:** Qiang Gao, Nanxi Chen, Baoyi Li, Menghang Zu, Ya Ma, Haiting Xu, Zhenhua Zhu, Rui L. Reis, Subhas C. Kundu, Bo Xiao

**Affiliations:** 1 State Key Laboratory of Resource Insects, College of Sericulture, Textile, and Biomass Sciences, Southwest University, Beibei, Chongqing, 400715 China; 2 State Key Laboratory of Southwestern Chinese Medicine Resources, Pharmacy School, Chengdu University of Traditional Chinese Medicine, Chengdu, Sichuan 611137 China; 3 Department of Gastroenterology, The First Affiliated Hospital of Nanchang University, Nanchang, Jiangxi 330006 China; 4 3Bs Research Group, I3Bs — Research Institute on Biomaterials, Biodegradables and Biomimetics, Headquarters of the European Institute of Excellence on Tissue Engineering and Regenerative Medicine, University of Minho, AvePark, Barco, Guimarães 4805-017 Portugal; 5 ICVS/3B’s-PT Government Associate Laboratory, AvePark, Braga, Guimarães Portugal

**Keywords:** Natural nanomedicine, Green preparation, Oral route, Galactose-mediated endocytosis, Liver cancer

## Abstract

The clinical application of conventional medications for hepatocellular carcinoma treatment has been severely restricted by their adverse effects and unsatisfactory therapeutic effectiveness. Inspired by the concept of ‘medicine food homology’, we extracted and purified natural exosome-like lipid nanoparticles (LNPs) from black mulberry (*Morus nigra* L.) leaves. The obtained MLNPs possessed a desirable hydrodynamic particle size (162.1 nm), a uniform size distribution (polydispersity index = 0.025), and a negative surface charge (-26.6 mv). These natural LNPs were rich in glycolipids, functional proteins, and active small molecules (e.g., rutin and quercetin 3-*O*-glucoside). In vitro experiments revealed that MLNPs were preferentially internalized by liver tumor cell lines *via* galactose receptor-mediated endocytosis, increased intracellular oxidative stress, and triggered mitochondrial damage, resulting in suppressing the viability, migration, and invasion of these cells. Importantly, in vivo investigations suggested that oral MLNPs entered into the circulatory system mainly through the jejunum and colon, and they exhibited negligible adverse effects and superior anti-liver tumor outcomes through direct tumor killing and intestinal microbiota modulation. These findings collectively demonstrate the potential of MLNPs as a natural, safe, and robust nanomedicine for oral treatment of hepatocellular carcinoma.

**Graphic abstract:**

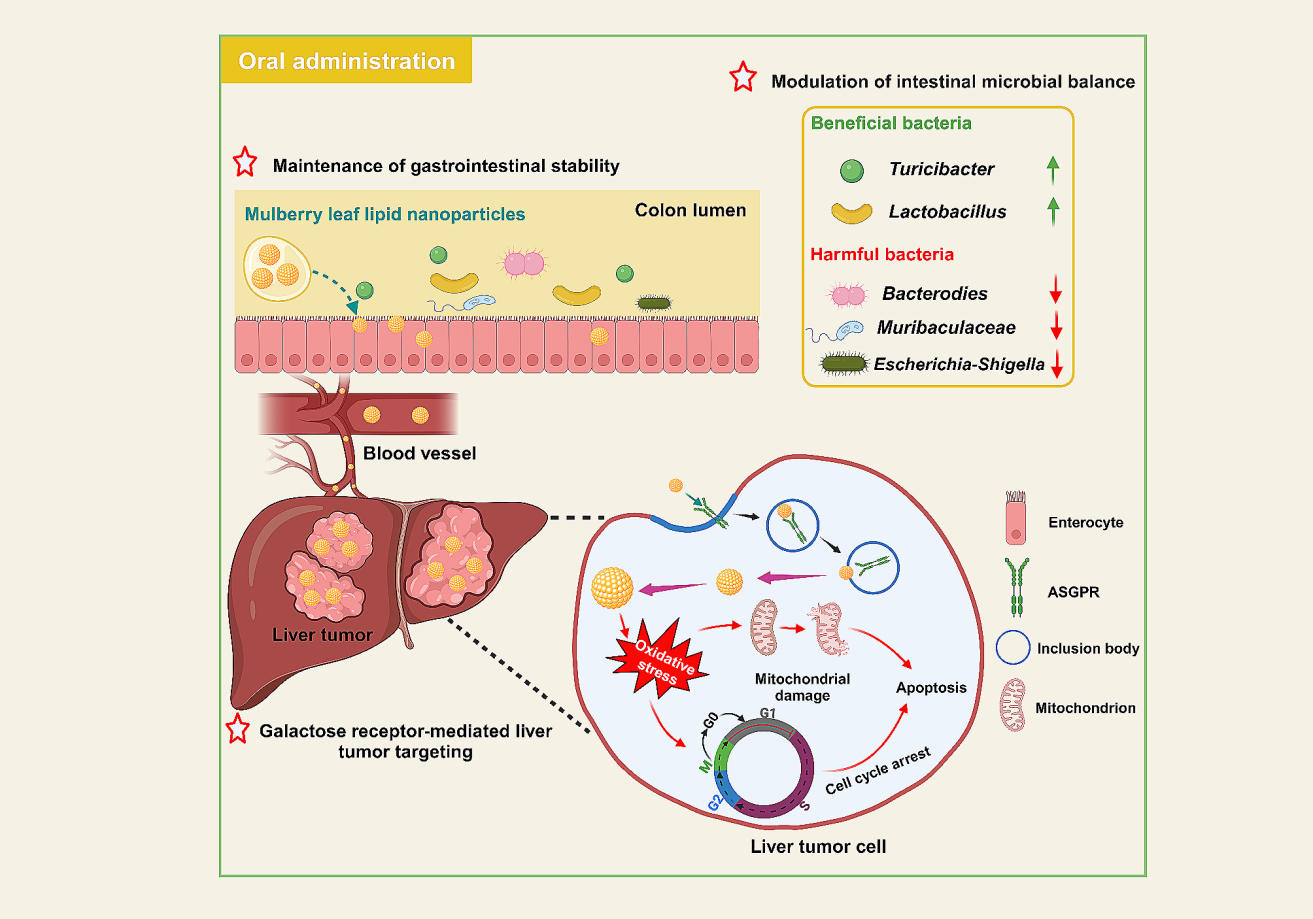

**Supplementary Information:**

The online version contains supplementary material available at 10.1186/s12951-023-02286-3.

## Introduction

Hepatocellular carcinoma (HCC) represents the predominant histological subtype of liver cancer, comprising approximately 90% of all cases [1]. It is the third most common cause of cancer-related deaths worldwide, which has garnered global attention due to its increasing morbidity and mortality [2]. Surgical treatments (e.g., resection, transplantation, and transcatheter arterial chemoembolization) have succeeded initially in clinical HCC treatment [3–5]. However, the inherent limitations within these therapeutic modalities, including decreased patient adherence, tumor recurrence, and high mortality rates, constitute substantial impediments to their pragmatic application [6–8]. Meanwhile, clinical chemotherapeutic drugs meet the challenges of low response rates, serious adverse effects, and limited lifespan extension, making the urgent requirement for novel HCC treatment platforms.

Exosomes are one of the categories of extracellular vesicles (EVs) with diameters ranging from approximately 40 to 160 nm (average ~ 100 nm) [9], which have attracted enormous attention from scientists in the field of disease detection, drug delivery, and tissue regeneration. Despite the widespread utilization of these exosomes across various disciplines, their extended medical applications remain constrained by inefficient preparation methods, substantial biological hazards, potential adverse immune reactions, and the formidable economic challenges associated with mass production [10–14]. In contrast to exosomes originating from animal sources, exosome-like lipid nanoparticles (LNPs) derived from edible plants exhibit many advantageous attributes, encompassing substantial scalability in production, heightened biosafety, cost-effectiveness, and stability in the gastrointestinal tract (GIT) [15–18]. Recently, we extracted natural LNPs from tea leaves and purified them using gradient ultracentrifugation. It was found that these LNPs could be efficiently internalized by activated macrophages, scavenge intracellular reactive oxygen species (ROS), and down-regulate inflammatory responses. Subsequent murine experiments elucidated that oral administration of tea leaf-derived LNPs reinstated compromised colonic barriers, mitigated colonic inflammation, and rebalanced intestinal microbiota. These outcomes collectively contributed to the prophylaxis and therapeutic intervention against ulcerative colitis and colitis-associated colorectal cancer [19]. Moreover, plant-derived LNPs exhibit favorable efficacies in treating various liver diseases. For example, natural LNPs from ginger were found to activate nuclear factor erythroid 2-related factor 2, up-regulate the expression levels of genes related to liver detoxification and antioxidant activity, and suppress ROS production, thereby exerting a positive protective effect against alcohol-induced liver injury in mice [20]. Another study found that LNPs extracted from *Asparagus cochinchinensis* were taken up by HepG2 cells mainly *via* phagocytosis and exerted an anti-tumor effect through an apoptosis-inducing pathway. Since these LNPs were quickly eliminated from the circulatory system, their surfaces were functionalized by polyethylene glycol to prolong the circulation time and increase the accumulation in the liver tumors, eventually inducing the effective tumor growth retardation without adverse effects [21].

**Scheme 1:**
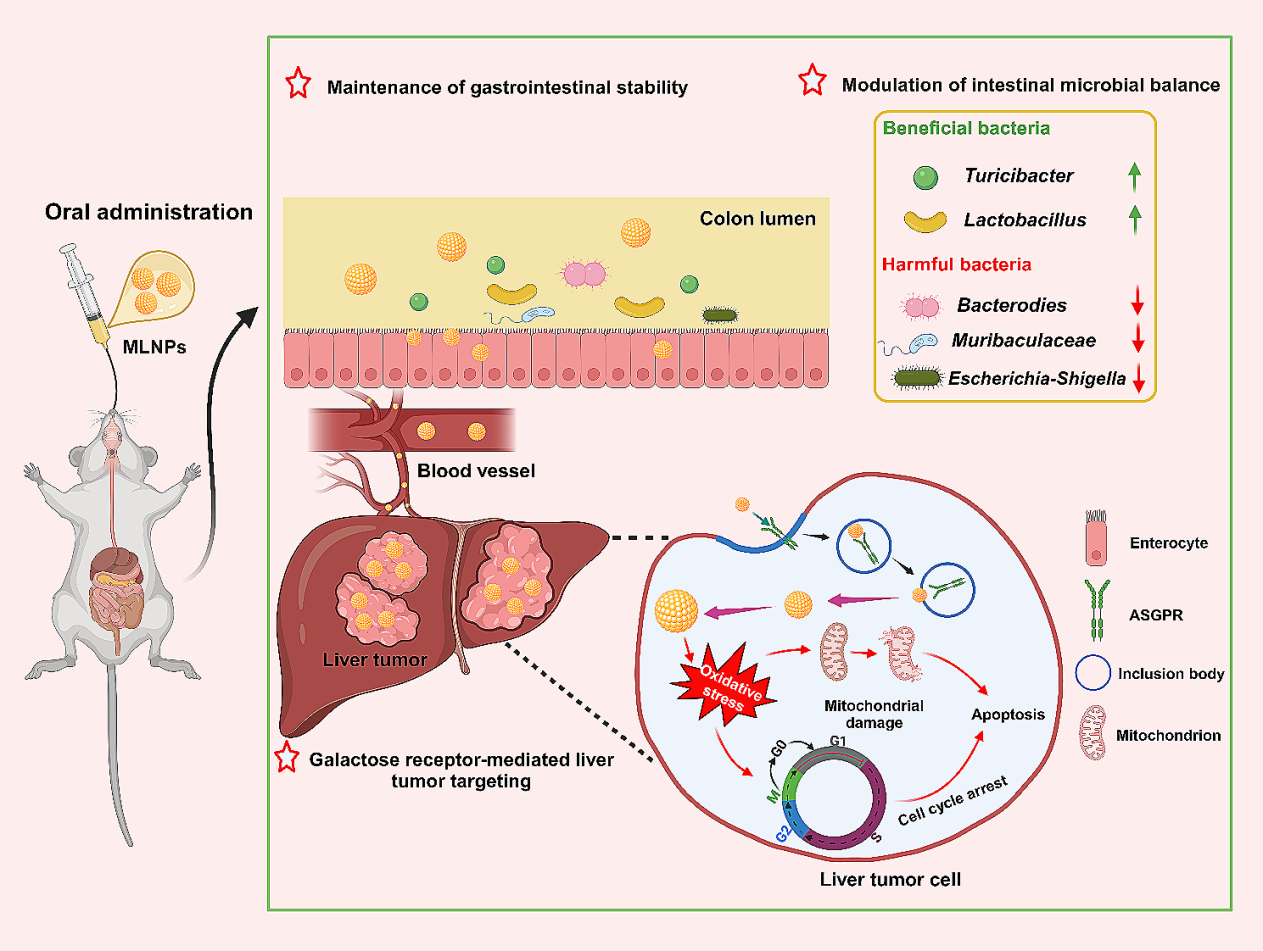
Schematic illustration of the transportation and therapeutic mechanism of MLNPs against HCC. After oral administration, MLNPs maintained stability during their passage through the GIT and modulated intestinal microbiota. The superior property effectively facilitated their unimpeded transit from the GIT into the hepatic vasculature *via* systemic circulation, ensuring comprehensive liver targeting. Subsequently, MLNPs underwent cellular internalization by liver tumor cells through the galactose-mediated endocytic processes. This intricate internalization mechanism further facilitated the efficacious eradication of Hepa1-6 cells by heightening intracellular oxidative stress, provoking mitochondrial damage, and inducing cell cycle arrest

Mulberry leaves have traditionally been employed as a feed source for silkworms, imparting a reservoir of diverse bioactive constituents, including alkaloids, polysaccharides, anthocyanins, and flavonoids [22, 23]. Thus, they are recommended as natural herbs with antioxidant, hypoglycemic, antibacterial, and anti-inflammatory properties [24]. Our group has maintained a steadfast commitment to developing natural and eco-friendly oral nanomedicines, yielding noteworthy outcomes in the therapeutic management of a spectrum of inflammatory and malignant disorders [25, 26]. Herein, we extracted LNPs from *Morus nigra* L. leaves (referred to as MLNPs) and systematically characterized their physicochemical properties. Subsequently, we investigated their internalization efficiencies by liver tumor cells, elucidated the underlying liver tumor-targeting and anti-tumor mechanisms, and assessed their therapeutic efficacy against HCC (Scheme 1). Finally, the biological safety of MLNPs was comparably evaluated by intravenous injection and oral administration.

## Materials and methods

### Materials

Fresh *Morus nigra L.* leaves were obtained from Xinjiang Hotan Silkworm Science Research Institute (Xinjiang, China). Sucrose was supplied by Adamas (Shanghai, China). Phosphate buffered saline (PBS) was purchased from Service Biotechnology Co., Ltd. (Wuhan, China). Methylthiazolyldiphenyl-tetrazolium bromide (MTT) and total bilirubin (TBIL) assay kits were from Solar Biotechnology Co., Ltd (Beijing, China). Urea nitrogen activity assay kits (BUN), creatinine assay kits (CRE), aspartate aminotransferase assay kits (AST), *γ*-glutamyl transferase (GGT) assay kits, and alanine aminotransferase assay kits (ALT) were supplied by Nanjing Jiancheng Bioengineering Institute (Jiangsu, China). Dimethyl sulfoxide (DMSO), Triton X-100, cell cycle analysis kits, 3,3ʹ-dioctadecyloxacarbocyanine perchlorate (DiO), 2-(4-amidinophenyl)-6-indolecar-bamidine dihydrochloride (DAPI), BCA protein assay kits, ROS assay kits, enhanced mitochondrial membrane potential assay kits with JC-1 assay kits, Hoechst 33,342, and terminal deoxynucleotidyl transferase-mediated dUTP-biotin nick end labeling (TUNEL) apoptosis assay kits were purchased from Beyotime Institute of Biotechnology (Shanghai, China). Rhodamine phalloidin and 1,1ʹ-dioctadecyl-3,3,3ʹ,3ʹ-tetramethylindotricarbocyanine iodide (DiR) were obtained from Thermo Fisher Scientific (Waltham, MA, USA).

### Isolation and purification of MLNPs

*Morus nigra L.* leaves were washed with water, soaked in PBS, and blended at a high speed for 10 min to obtain leaf juice. The obtained leaf juice was centrifuged at 3,000 × *g* (20 min) and 10,000 × *g* (40 min) sequentially to precipitate large cell fragments. The supernatant was collected and centrifuged at 15,000 × *g* for 60 min to obtain the precipitate. After that, the precipitate was diluted with PBS, received ultrasound (200 W), and centrifuged at 15,000 × *g* for 60 min through sucrose density gradients (sucrose gradients of 8%, 30%, 45%, and 60%, w/v). Eventually, MLNPs were collected from the 30/45% sucrose interface, quantified using a BCA assay kit, and stored at -80 °C.

### In vitro anti‑tumor activity of MLNPs

The anti-tumor activity of MLNPs was evaluated using MTT assay. HepG2, CT-26, Hepa1-6, 4T1, A549, L929, and MC3T3-E1 cells were seeded at a density of 1 × 10^4^ cells/well in 96-well plates and incubated overnight. Cells were co-incubated with MLNPs (1, 2, 5, 10, 20, 50, and 100 μg/mL) for 24 and 48 h, respectively. Cells were washed with PBS and incubated with MTT solution (0.5 mg/mL) at 37 °C for 4 h. The supernatant was discarded, and DMSO (100 μL) was added to each well before measuring spectrophotometrically at 570 nm.

### In vivo bio-distribution of MLNPs

6-Week-old female C57BL/6J mice were obtained from Chongqing Byrness Weil Biotechnology Co. Ltd. (Chongqing, China). Mice protocols were approved by the Institutional Animal Care and Use Committee of Southwest University. To establish HCC mouse model, mice were intraperitoneally injected with diethylnitrosamine (DEN) solution (20 mg/kg) and received the treatment of *N*-nitrosomorpholine (NMOR, 80 ppm)-contained drinking water for 24 weeks. Mice were orally administered DiR-MLNPs at a concentration of 5 mg protein/kg per mouse. At predetermined time points (12, 24, 48, and 72 h), mice were sacrificed, and the major organs and GITs were isolated and imaged using an IVIS spectrum imaging system (PerkinElmer/Caliper LifeSciences, Hopkinton, MA, USA).

In vivo **therapeutic outcome of MLNPs against HCC**

The HCC mouse model was established following the above protocol. The treatment groups were treated with MLNPs at a dose of either 2.5 mg/kg (L) or 5 mg/kg (H) every 3 days *via* the oral route. Mice were monitored for changes in their status, body weight, and survival rate throughout the experiments. After administration for 5 dosages, mice were sacrificed, and their major organs, blood samples, and feces were collected for analysis.

### Statistical analysis

Data were presented as means ± standard error of the mean (S.E.M.). Statistical analysis was carried out using Student’s *t*-test or one-way ANOVA. Statistical significance was represented by *p < 0.05, **p < 0.01, and ***p < 0.001.

## Results and discussion

### Physicochemical characterization of MLNPs

**Fig. 1:**
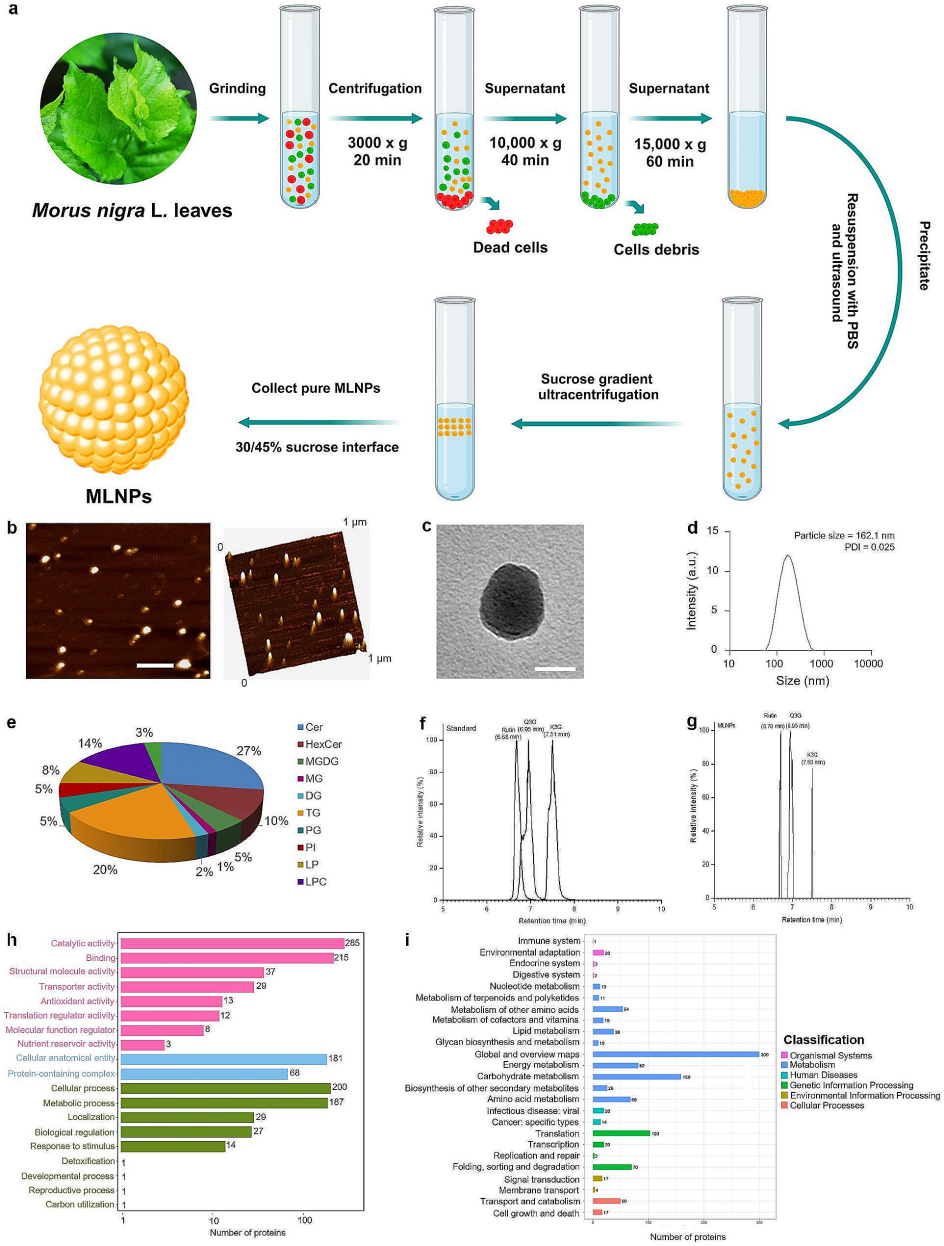
Physicochemical characterizations of MLNPs. (**a**) Extraction of MLNPs from *Morus nigra* L leaves. (**b**) AFM images (scale bar = 200 nm), (**c**) TEM images (scale bar = 50 nm), (**d**) hydrodynamic particle size distribution, and (**e**) lipid compositions of MLNPs. Chromatograms of (**f**) standard and (**g**) MLNPs. (**h**) GO secondary classification statistical chart and (**i**) KEGG annotated statistical chart of MLNPs

MLNPs were extracted from fresh *Morus nigra* L. leaf juice and purified through differential centrifugation and sucrose density gradient ultracentrifugation (Fig. 1a). Driven by the sucrose gradients, MLNPs were mainly distributed in the sucrose density layer of 30/45%. Atomic force microscopy (AFM) and transmission electron microscopy (TEM) revealed that MLNPs appeared as exosome-like spherical particles with a diameter of approximately 100 nm following dehydration (Fig. 1b, c). The further analysis of dynamic light scattering (DLS) unveiled that MLNPs possessed a hydrodynamic particle size of 162.1 nm and exhibited an exquisitely uniform size distribution (polydispersity index; PDI = 0.025), as shown in Fig. 1d. The mean diameters of MLNPs obtained by AFM and TEM were significantly smaller than that determined by DLS, probably attributing to their shrinkage before AFM/TEM and swell during DLS tests.

The lipidomic analysis revealed that MLNPs were predominantly comprised of ceramides (Cer, 27%), triglycerides (TG, 20%), hemolytic phosphatidylcholine (LPC, 14%), and hexose ceramides (HexCer, 10%). These constituents significantly contributed to the stability of MLNPs while orchestrating the regulation of diverse physiological processes, encompassing macrophage activation, tissue remodeling, and wound healing. We found that hemolytic phospholipids were present in MLNPs, which could facilitate lipid fusion and enhance the transfer of vesicular contents to cells [27]. Meanwhile, MLNPs were found to contain 5.0% of monogalactodiglycerol (MGDG) (Fig. 1e), which exhibited a specific affinity towards the asialoglycoprotein receptor (ASGPR) over-expressed on the surface of liver tumor cells, thereby facilitating efficient live tumor-targeted delivery of bioactive molecules [28]. As reported, mulberry leaves harbored a plethora of small active molecules, encompassing an extensive range of flavonoids, polyphenols, polysaccharides, alkaloids, and other small compounds. Flavonoids in nanovesicles were determined using an ultra-performance liquid chromatography system and comparison with the mulberry metabolomics database (MMHub) [29, 30]. It was detected that MLNPs contained 3 primary flavonoids: rutin, quercetin 3-*O*-glucoside (Q3G), and kaempferol-3-*O*-glucoside (K3G) (Fig. 1f, g). The Gene Ontology (GO) functional database annotated a total of 1,312 genes for comprehensive functional classification, of which 602 genes participate in various molecular functions distributed across 8 distinct categories, notably inclusive of catalytic activity and binding. Additionally, 561 genes were identified to be involved in the biochemical processes, which were further divided into 9 seed functions, with cellular and metabolic processes being the primary functions. Eventually, 249 genes were found to be associated with cellular components consisting solely of cellular anatomical entities and protein-containing complexes (Fig. 1h).

KEGG pathway analysis revealed that 34 proteins in MLNPs were associated with human diseases and liver cancer-associated signaling pathways (Fig. 1i). The growth factors (TGF-*α*, TGF-*β*, IGF-II, and HGF) and Wnt signaling pathway are reactivated during tissue regeneration, cell renewal, and certain pathological conditions (e.g., premalignant disease and cancer). The Wnt/*β*-catenin protein pathway was predominantly quiescent in the mature and healthy liver [31]. A significant proportion of liver tumors harbored mutations in genes encoding the critical components of the Wnt/*β*-catenin protein signaling pathway. These findings provide an insight into the molecular mechanism by which mulberry nanovesicles inhibit hepatoma cells.

### In vitro anti‑tumor activities of MLNPs

**Fig. 2:**
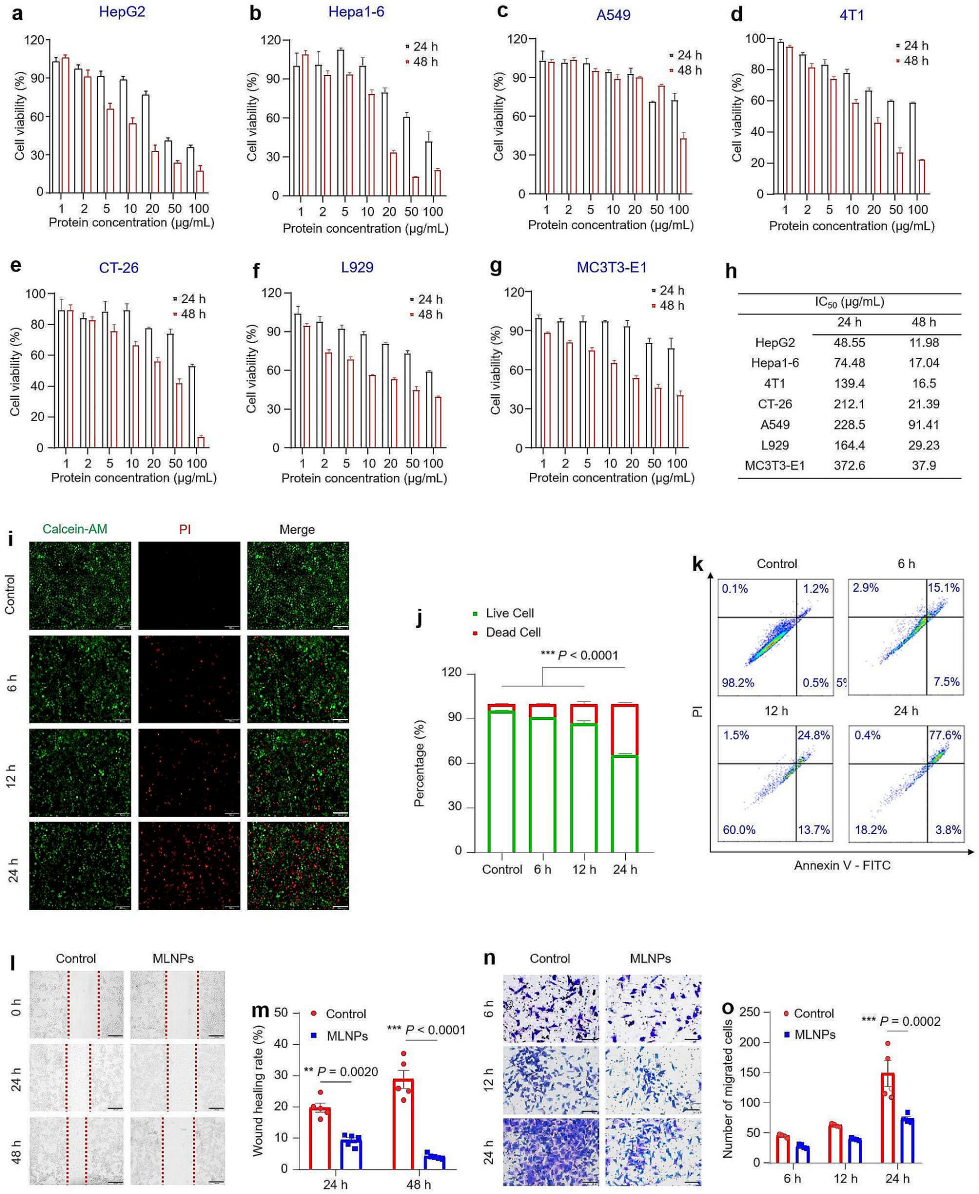
In vitro anti‑tumor activities of MLNPs. MTT assay was used to assess the potential toxicity of MLNPs against (**a**) HepG2, (**b**) Hepa1-6, (**c**) A549, (**d**) 4T1, (**e**) CT26, (**f**) L929, and (**g**) MC3T3-E1 cells after co-incubation for 24 and 48 h, respectively. Each point represents the mean ± S.E.M. (n = 5). (**h**) The IC_50_ values of MLNPs against different cell lines. Each point represents the mean ± S.E.M. (n = 5). (**i**) Fluorescence images of Hepa1-6 cells stained with Calcein-AM/PI. Live cells were stained green with calcein-AM, and dead cells were stained red with PI (Scale bar = 200 μm). (**j**) Quantitative analysis of the fluorescence intensity of live or dead cells. Each point represents the mean ± S.E.M. (n = 5; *p < 0.05, **p < 0.01, and ***p < 0.001). (**k**) Apoptosis effect of Hepa1-6 cells with the treatment of MLNPs for 6, 12, and 24 h, respectively. (**l**) Migration of Hepa1-6 cells with or without the treatment of MLNPs for 24 and 48 h, respectively (Scale bar = 200 μm). (**m**) Wound healing rates of Hepa1-6 cells in the presence or absence of MLNPs using ImageJ software. Each point represents the mean ± S.E.M. (n = 5; *p < 0.05, **p < 0.01, and ***p < 0.001). (**n**) The transwell migration capacity of Hepa1-6 cells with the treatment of MLNPs for 6, 12, and 24 h, respectively (Scale bar = 100 μm). (**o**) Cell counts of transwell migration at 6, 12, and 24 h using ImageJ software. Each point represents the mean ± S.E.M. (n = 4; *p < 0.05, **p < 0.01, and ***p < 0.001)

MTT assay was used to evaluate in vitro inhibitory potential of MLNPs against various tumor and healthy cell lines. The dose-dependent and time-dependent anti-proliferative effects of MLNPs were observed across all 6 cell lines (Fig. 2a-g). Amongst HepG2, Hepa1-6, CT-26, 4T1, and A549 cells, MLNPs exhibited maximum cytotoxicity towards HepG2 and Hepa1-6 cells, and IC_50_ values of the MLNP-treated HepG2 and Hepa1-6 cells were 48.6 and 74.5 μg/mL after a 24-hour exposure, and 12.0 and 17.0 μg/mL after 48 h, respectively (Fig. 2g). The strongest anti-proliferative effect on HepG2 and Hepa1-6 cells might be ascribed to the presence of galactose end group-contained glycolipids in the MLNPs that specifically target hepatoma cells. These results demonstrate that MLNPs have a promising potential for HCC treatment. Notably, MLNPs demonstrated relatively lower toxicities against healthy cells than hepatoma cells (Fig. 2f, g), suggesting their good biocompatibility.

Next, we used live/dead cell double staining to verify the toxicity of MLNPs to hepatoma cells. Calcein-AM enters living cells and emits green fluorescence after being broken down by esterases, while PI only enters dead cells and emits red fluorescence when binds to DNA [32]. As depicted in Fig. 2i, Hepa1-6 cells demonstrated a discernible time-dependent augmentation in red fluorescence intensities subsequent to co-incubation with MLNPs for 6, 12, and 24 h, in contrast to the control group. The quantitative results revealed a survival rate of approximately 60% of cells following a 24-hour treatment with MLNPs (Fig. 2j), a finding in concordance with the results obtained from the MTT assay. Moreover, Annexin V-FITC/PI staining revealed that advanced apoptosis or necrosis occurred in 77.6% of Hepa1-6 cells receiving the treatment of MLNPs at 24 h (Fig. 2k). Tumor cell metastasis involves migration and invasion, which causes over 90% cancer deaths [33, 34]. To evaluate the impacts of MLNPs on tumor invasion and metastasis, we conducted cell scratch and migration assays. It was observed that MLNPs significantly reduced wound healing rates to 9.3% and 4.1% after co-incubation for 24 and 48 h, respectively (Fig. 2l, m), indicating their substantial capacity to inhibit the migration of hepatoma cells. In alignment with the observations from the cell scratch assay, it was noteworthy that MLNPs showed a time-dependent influence in impeding the invasion and metastasis of tumor cells within a defined temporal range (Fig. 2n, o). Therefore, we posit that MLNPs could be exploited as a promising natural nanomedicine for combating metastatic liver cancers.

### In vitro cellular uptake and anti‑tumor mechanism of MLNPs

**Fig. 3:**
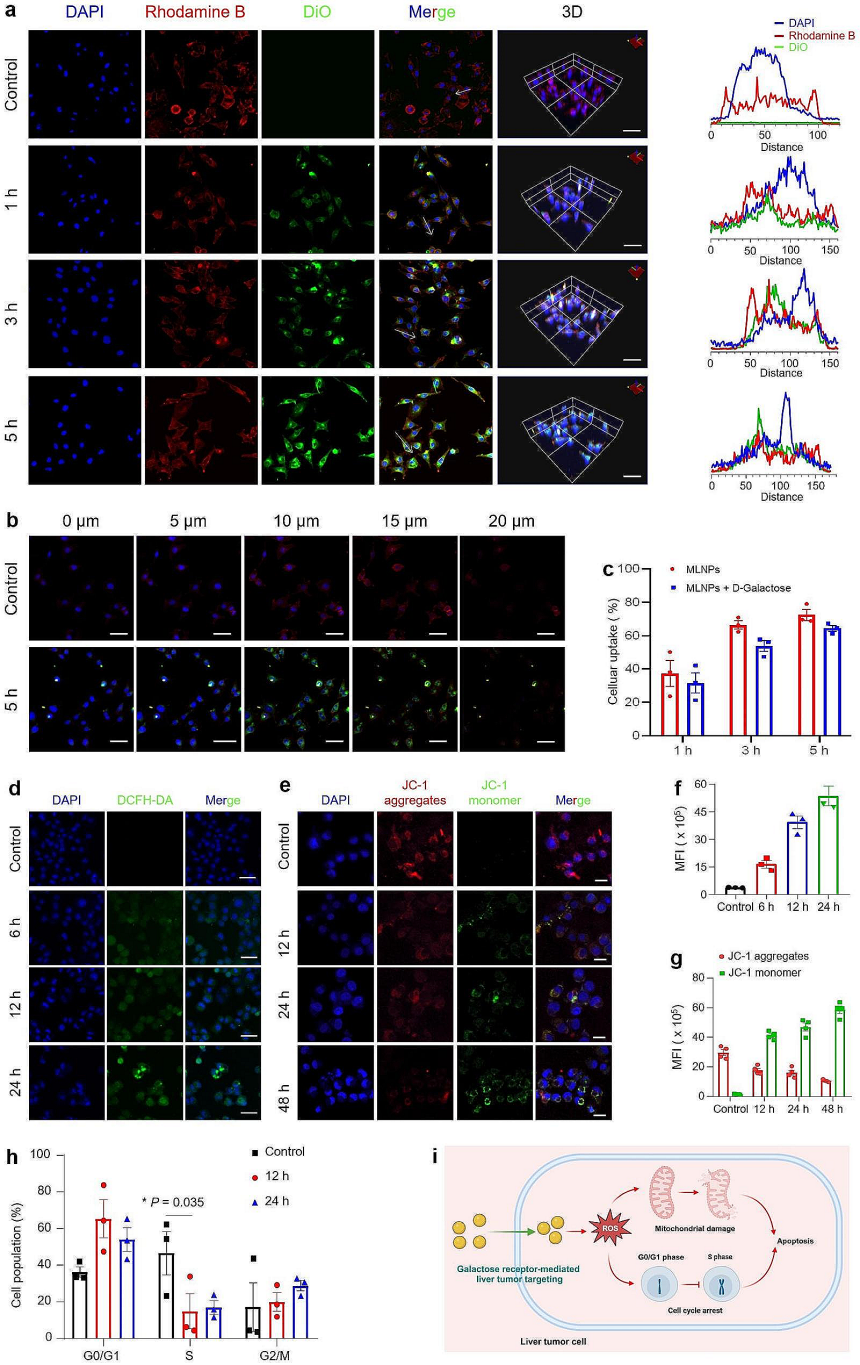
In vitro cellular uptake and antitumor mechanism of MLNPs. (**a**) CLSM images and fluorescence distribution profiles (Scale bar = 50 μm). (**b**) CLSM cross-section images of 5-layered cellular uptake of DiO-labeled MLNPs (green) after incubation for 5 h. Hepa1-6 cells were labeled with DAPI (blue) and Rhodamine phalloidin (red) (Scale bar = 50 μm). (**c**) Percentages of DiO-labeled MLNPs internalized by Hepa1-6 cells for 1, 3, and 5 h, respectively. Each point represents the mean ± S.E.M. (n = 3). (**d**) CLSM images and (**f**) quantification of ROS changes in Hepa1-6 cells labeled with Hoechst 33,342 (blue) after co-incubation with MLNPs for 6, 12, and 24 h, respectively (Scale bar = 50 μm). Each point represents the mean ± S.E.M. (n = 3). (**e**) CLSM images and (**g**) quantification of JC-1 and Hoechst 33,342 stained Hepa1-6 cells after co-incubation with MLNPs for 12, 24, and 48 h, respectively (Scale bar = 20 μm). Each point represents the mean ± S.E.M. (n = 4). (**h**) Cell cycle analysis of Hepa1-6 cells after co-incubation with MLNPs for 12 or 24 h by FCM. Each point represents the mean ± S.E.M. (n = 3; *p < 0.05). (**i**) Schematic illustration of the pro-apoptotic mechanism of MLNPs against liver tumor cells

The ability of nanomedicines to be internalized by targeted cells is a crucial factor that affects its efficacy [35]. Thus, we assessed the cellular uptake profiles of MLNPs by hepatoma cells. Since MLNPs lacked fluorescence but possessed lipophilic properties, they were labeled with a lipophilic dye (DiO). It was detected that green fluorescence signals within Hepa1-6 cells gradually increased with their elongated incubation with MLNPs, which overlapped with red fluorescence of the cytoskeleton (Fig. 3a-c). This observation substantiates the effective internalization of MLNPs by Hepa1-6 cells, with predominant distribution observed within the cytoplasm. Following co-incubation periods of 1, 3, and 5 h with MLNPs, Hepa1-6 cells exhibited uptake percentages of 37.3%, 66.0%, and 72.5%, respectively (Fig. 3c). In contrast, L929 cells displayed notably lower uptake percentages, measuring 4.7%, 20.0%, and 30.8%, respectively (Fig. S1). Furthermore, our investigation revealed decrements in the uptake percentages of MLNPs by Hepa1-6 cells following co-culture with free galactose, with reductions of 5.8%, 12.7%, and 8.1% observed after 1, 3, and 5 h, respectively. This underscores the robust liver tumor-targeting capacity of MLNPs through the galactose receptor-mediated approach.

Intracellular ROS can induce oxidative damage to DNA, proteins, and lipids, ultimately leading to the apoptosis of tumor cells [36]. We thus investigated the ROS levels in Hepa1-6 cells receiving the treatment of MLNPs. It was detected that the control cells (without MLNP treatment) showed negligible green fluorescence signals. On the contrary, a gradual augmentation in the green fluorescence intensity was observed within Hepa1-6 cells receiving the treatment of MLNPs for different periods (6, 12, and 24 h), reflecting that MLNPs could effectively induce ROS generation in hepatoma cells (Fig. 3d, f). Loss of mitochondrial membrane potential is a hallmark event during apoptosis [37]. JC-1 probe has been commonly applied to detect the variations of mitochondrial membrane potential. In principle, mitochondrial membrane potential will decrease in apoptotic cells, and JC-1 molecules cannot be enriched in their mitochondrial matrix and exist as monomers that excite green fluorescence under the FITC channel of confocal microscope [38]. As seen in Fig. 3e, g, a discernible attenuation in red fluorescence and a concurrent augmentation in green fluorescence were observed following the administration of MLNPs. These findings signify the capacity of these naturally derived MLNPs to induce structural damage to the mitochondrial membrane. Furthermore, PI staining was conducted to explore the capacity of MLNPs in inducing cell cycle arrest. Figure 3h illustrated that after the treatment with MLNPs for 12 and 24 h, there was a notable elevation in the percentage of cells in the G0/G1 phase, concomitant with a decrement in the proportion of cells in the S phase. These observations demonstrate that MLNPs can block the cell cycle of Hepa1-6 cells and lead them to stagnate in the G0/G1 phase (Fig. 3i).

### In vivo biosafety evaluation of MLNPs

Biosafety is a critical factor for the clinical translation of nanomedicines [39]. We thus evaluated the in vivo biosafety of MLNPs *via* intravenous injection and oral administration. It was found that MLNPs exhibited excellent hemocompatibility compared with the positive control group, as evidenced by the absence of blood circulation disorders or significant hemolysis at any dosages (Fig. S2a, b). The body weight of mice in each group remained stable without any significant decline (Fig. S3a). As depicted in Fig. S3b-h, oral administration of MLNPs demonstrated no observable deviations in organ indices (specifically liver and kidney) and biochemical markers when compared to the healthy group. Conversely, the intravenous administration of MLNPs resulted in a notable increase in AST levels and a concurrent reduction in TBIL and GGT levels (AST, TBIL, and GGT being pivotal markers for assessing liver function). These findings imply a potential hepatic toxicity associated with intravenous administration of MLNPs, highlighting the relative biosafety of oral MLNPs. ALT and AST are present in the heart, which will enter the blood stream from the damaged heart tissues. These two parameters did not significantly increase after oral administration in comparison with the control group (Fig. S3c, d). In terms of renal function, we found that creatinine (CRE) and blood urea nitrogen (BUN), which reflect kidney function, showed no obvious changes among these three groups (Fig. S3g, h). These results demonstrate that oral MLNPs exert negligible toxicity to the heart and kidney. In addition, the blood parameters in all groups were within the normal ranges, indicating that MLNPs did not cause hematological toxicity (Fig. S4). The tissue morphology of the five principle organs from the treatment groups exhibited no significant deviation from that of the healthy control group (Fig. S5), affirming the exceptional biosafety profile of MLNPs after oral administration.

### In vivo bio‑distribution and liver targeting of MLNPs

**Fig. 4:**
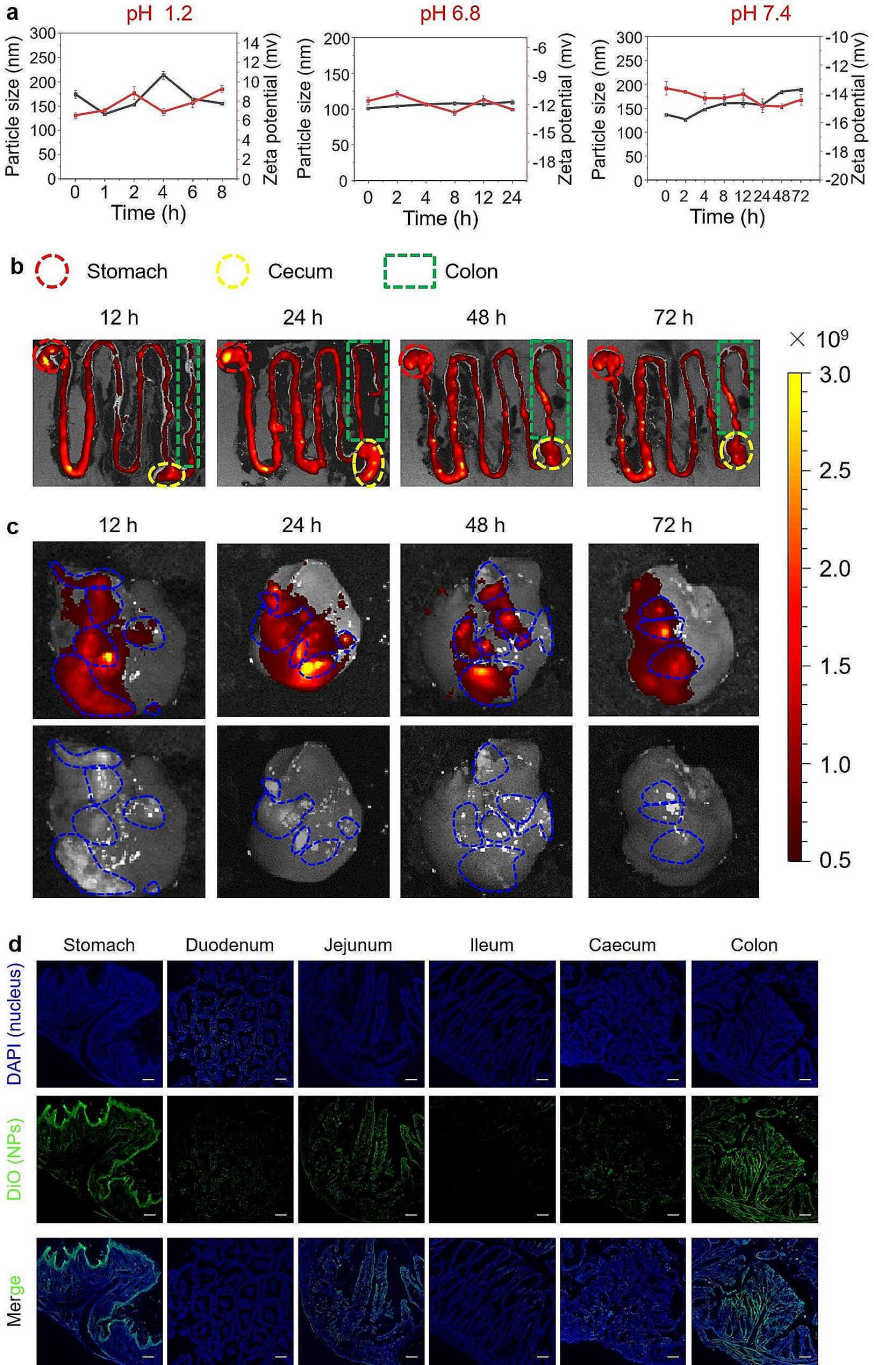
In vivo biodistribution and liver targeting profiles of MLNPs. (**a**) Stabilities of MLNPs in the simulated gastric, small intestinal, and colonic fluids. (**b**) Ex vivo fluorescence images of the GIT in the HCC mouse model receiving oral administration of DiR-MLNPs at different time points (12, 24, 48, and 72 h). (**c**) Ex vivo tumor-targeted fluorescence images and bio-distribution of DiR-labeled MLNPs in the orthotopic liver cancer mouse model. (**d**) Fluorescence images of the GIT sections from mice receiving oral administration of DiO-MLNPs (Scale bar = 100 μm). Each point represents the mean ± S.E.M. (n = 3)

The oral route is one of the most preferred approaches for drug delivery due to its convenience, cost-effectiveness, and high patient compliance [40]. The stability of therapeutics in the GIT is a crucial prerequisite for oral administration [41]. Thus, we investigated the stability of MLNPs in the simulated gastric, small intestinal, and colonic fluids. Figure 4a revealed that MLNPs possessed relatively stable particle sizes and surface charges during the incubation in different simulated solutions, suggesting their stability in the GIT. The robust preservation of the structural integrity of MLNPs ensured their unblemished journey from the GIT to the liver tumors. DiR is a common near-infrared fluorescent probe, which can bind to the phospholipid bilayer structure of LNPs. Consequently, we employed DiR as a labeling agent for MLNPs to track their biodistribution within HCC mouse model. It was observed that after oral administration, DiR-labeled MLNPs maintained in the GIT over 72 h (Fig. 4b). Strikingly, the fluorescence signals of MLNPs were perfectly overlapped with the liver tumors (Fig. 4c), demonstrating their intrinsic liver tumor-targeting capacity.

The fluorescence intensity emanating from DiR-labeled MLNPs reached its zenith within the GIT following 24 h of oral administration, as depicted in Fig. 4b. Subsequently, a detection was conducted to ascertain the specific absorption site within the GIT at this designated time point. Figure 4d showed that obvious green fluorescence signals (MLNPs) were detected in the stomach, jejunum, and colon. Moreover, these green signals were enriched in the epithelial layer of the stomach, while they were mainly present in the mucosa of the jejunum and colon. These observations suggest that MLNPs might not be absorbed in the stomach but in the jejunum and colon, where they enter the circulatory system and accumulate in the liver tumors *via* galactose receptor-mediated targeting.

### In vivo therapeutic outcomes of MLNPs against HCC

**Fig. 5:**
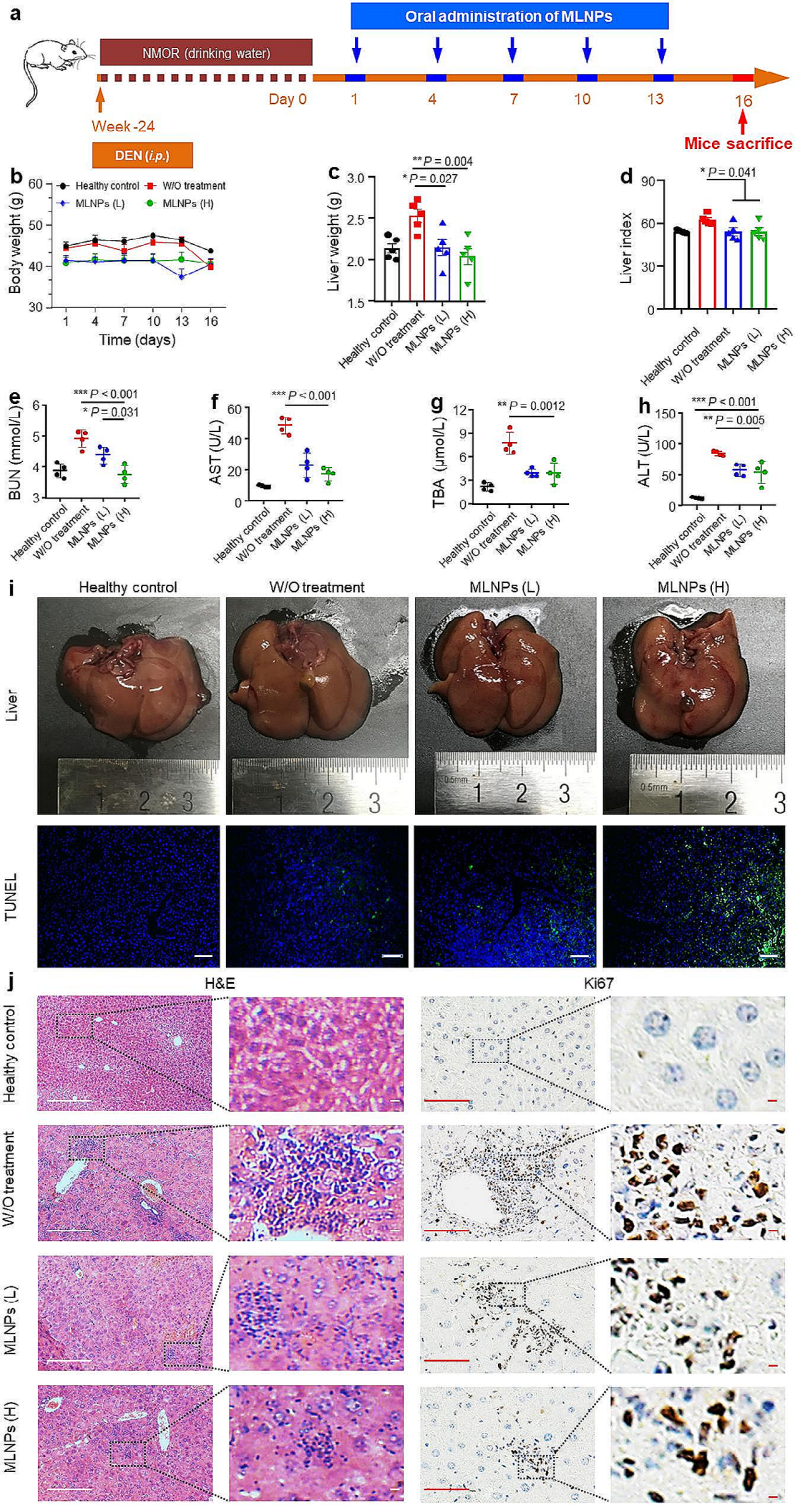
In vivo therapeutic outcomes of MLNPs against HCC. (**a**) Schematic diagram of the establishment process of orthotopic liver cancer mouse model and treatment process. (**b**) The body weight, (**c**) liver weight, and (**d**) organ index of orthotopic liver cancer mice with the treatment of MLNPs. Each point represents the mean ± S.E.M. (n = 5; *p < 0.05 and **p < 0.01). The amounts of (**e**) BUN, (**f**) AST, (**g**) TBA, and (**h**) ALT in the serum. Each point represents the mean ± S.E.M. (n = 4; *p < 0.05, **p < 0.01, and ***p < 0.001). (**i**) Representative digital photos, TUNEL (Scale bar = 100 μm), (**j**) H&E (Scale bar = 100 μm), and Ki67 (Scale bar = 100 μm) staining images of the liver tissues from mice receiving oral treatment of MLNPs. Each point represents the mean ± S.E.M. (n = 3)

The anti-liver tumor effect of MLNPs was assessed on the basis of the DEN/NMOR-induced orthotopic liver cancer mouse model, which could closely mimic human liver cancer. Following the establishment of an orthotopic HCC model, mice were subjected to oral administration of MLNPs on days 1, 4, 7, 10, and 13 (Fig. 5a). No apparent disparity in body weight was found among all mouse groups during the entire investigations (Fig. 5b). We further found that compared with the healthy control group, the control group (without treatments) possessed increased liver weight (Fig. 5c) and liver index (Fig. 5d). However, oral administration of MLNPs (low dose: 2.5 mg/kg; high dose: 5 mg/kg) led to significant reductions in liver weight and liver index. Moreover, treating MLNPs significantly improved the hepatic functions of mice with orthotopic liver tumors. Specifically, the group treated with MLNPs (L) demonstrated reductions of 1.1-, 2.1-, 2.0-, and 1.5-fold in the levels of BUN (Fig. 5e), AST (Fig. 5f), TBA (Fig. 5g), and ALT (Fig. 5h), respectively. In addition, we found that increasing the treatment amounts of MLNPs slightly decreased the liver function indices.

**Fig. 6:**
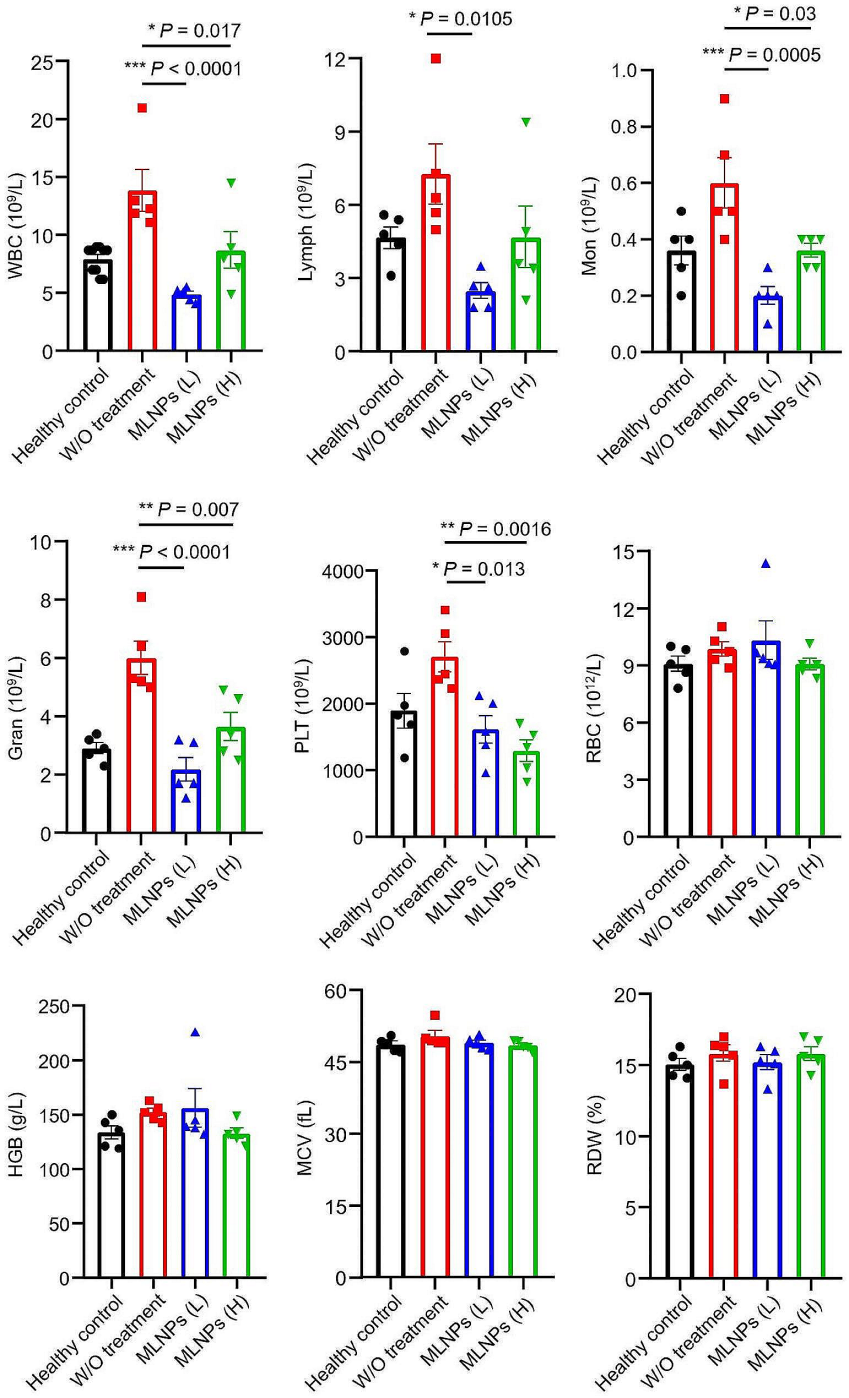
Complete blood count of the orthotopic liver cancer mice with oral treatment of MLNPs. Each point represents the mean ± S.E.M. (n = 5; *p < 0.05, **p < 0.01, and ***p < 0.001)

Next, we determined the morphologies of livers from various mouse groups. Figure 5i illustrated that the groups treated with MLNPs (L and H) exhibited markedly ameliorated hepatic histology, contrasting with the liver morphology observed in the untreated control group. We also found that the indices (Fig. S6a) and tissue morphologies (Fig. S6b) of other organs (heart, spleen, lung, and kidney) from the MLNP (L and H)-treated groups had no significant differences, compared with those from the healthy control group (Fig. S6a). The H&E staining images revealed the presence of early-stage primary liver tumor in the control group (without treatment), as evidenced by hepatocyte nuclear division and vacuolar deformation. Nevertheless, oral MLNPs (L and H) significantly decreased the presentation of primary liver tumors (Fig. 5j). The combination of TUNEL and Ki67 assays has been employed to label the apoptotic and proliferative profiles of cells in the tumor tissues, respectively. Remarkably, the livers from the MLNP (H)-treated group displayed the most intense TUNEL green fluorescence and the lowest Ki67 positivity (Fig. 5i, j). The hematological analysis revealed that the control group, devoid of any treatment, manifested heightened counts of white blood cells, encompassing lymphocytes, neutrophils, and monocytes. These counts surpassed those observed in the healthy control group, indicative of inflammatory responses provoked by liver tumors (Fig. 6). Interestingly, following treatment with low/high doses of MLNPs, the levels of various leukocytes exhibited significant reductions and approached normalcy. These findings suggest that oral treatment of MLNPs can efficiently retard the progression of liver tumors.

### Impacts of oral MLNPs on intestinal microbiota

The gut microbiome has recently been recognized as a major environmental factor in the pathobiology of many diseases. Throughout the preceding decades, numerous studies have consistently reported a discernible alteration in the composition of gut microbiota among patients with cirrhosis, which progressively becomes dysregulated as the development of liver diseases [42]. The liver receives approximately 70% of its blood supply from the intestine and transports various substances that are advantageous to intestinal nutrition and functions. Dysbiosis, characterized by quantitative and qualitative changes in gut microbiota, compromises the integrity of the intestinal barrier, leading to intestinal permeability and pathological bacterial translocations [43].

**Fig. 7:**
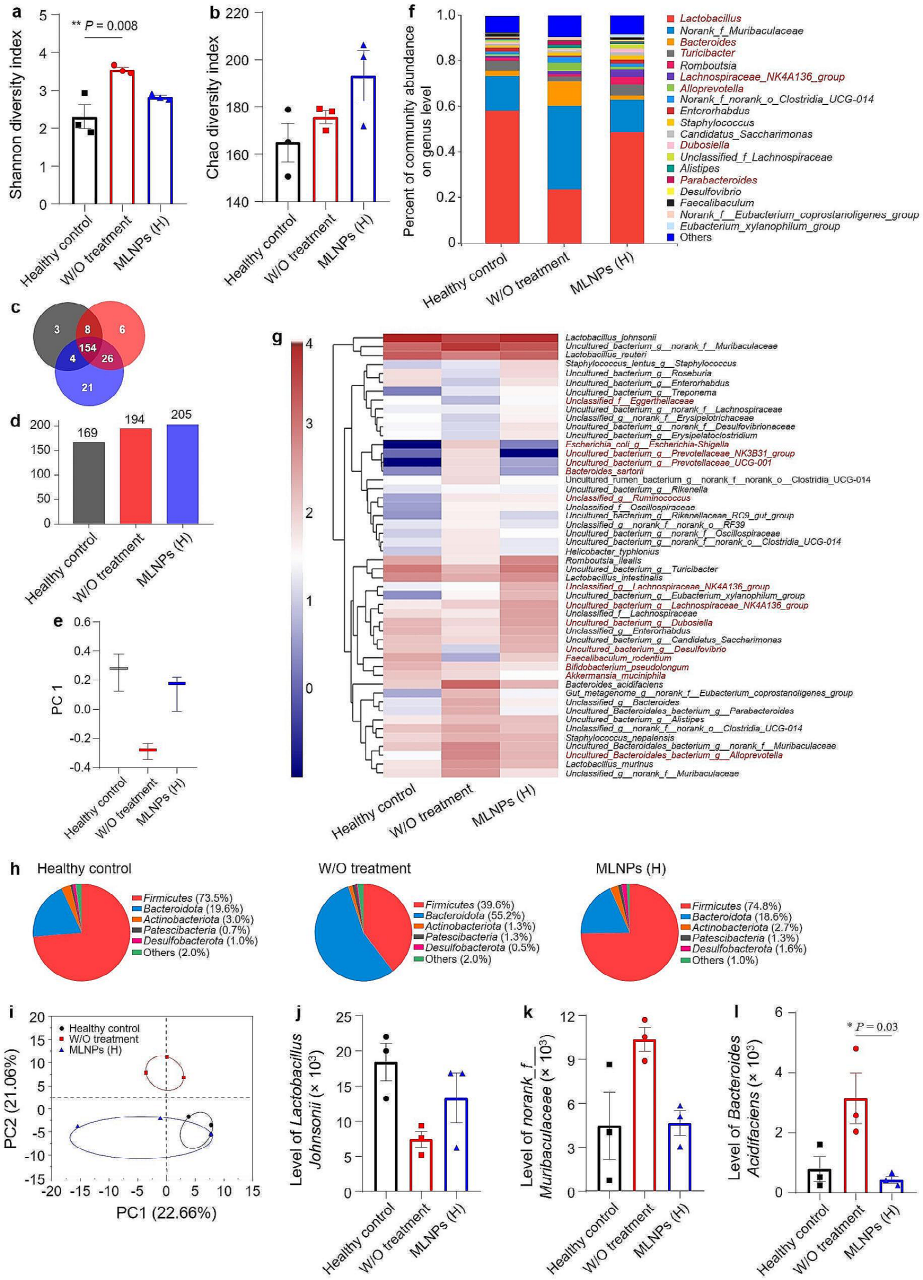
Evaluation of remodeling effects of MLNPs on the intestinal microbiota. *α*-Diversities were presented by box plots of the (**a**) Shannon index, and (**b**) Chao index. Each point represents the mean ± S.E.M. (n = 3; *p < 0.05 and **p < 0.01). (**c**) Venn diagram of common and unique bacterial species. (**d**) Total numbers of microbial species of mice in each group. (**e**) Dispersion of *β*-diversity from experiment outcome. (**f**) Percent of community abundance on the genus level of the intestinal microbiota. (**g**) Heatmap of relative abundance at the species level of the intestinal microbiota. (**h**) Microbial compositions of various mouse groups at the phylum level. (**i**) Principal coordinates analysis (PCoA) of the intestinal microbiota in each group. (**j**-**l**) Relative abundances of the typical beneficial and harmful bacteria in different treatment groups. Each point represents the mean ± S.E.M. (n = 3; *p < 0.05 and **p < 0.01)

To further elucidate the impacts of MLNPs on intestinal microbiota, we employed 16s RNA gene sequencing to comparatively investigate the gut microbiota compositions among different mouse groups. Compared with the healthy control group, DEN/NMOR treatment caused an increase in bacterial richness at the species level, accompanied by a corresponding elevation in the Shannon diversity index representing microbiota diversity. Oral MLNPs induced alterations in the microbiota diversities, as evidenced by changes in the Chao and Shannon diversity index (Fig. 7a, b). Venn diagram can visualize the number of common and unique species as well as similarities and overlaps between different groups. It showed that the MLNP (H)-treated group had a remarkable increase in colony formation with an impressive count of 205 strains (154 shared among all mouse groups and 21 unique to this group), which was consistent with the trend observed in the Chao Index of Richness (Fig. 7c, d). Discrete statistics from principal coordinates analysis (PCoA) illustrate how different groups are distributed along the PC1 axis. As presented in Fig. 7e, the distances among the healthy control group, the DEN/NMOR control group, and the MLNP (H)-treated group reflected the positive effect of MLNPs on microbiota remodeling, demonstrating that MLNPs could effectively reverse intestinal flora disorders caused by chemical carcinogens while maintaining stability.

Furthermore, the control group (without treatment) exhibited a higher relative abundance of detrimental bacteria (e.g., *Norank Muribaculaceae* and *Bacteroides*) when compared with the healthy control group (Fig. 7f). Following oral treatment of MLNPs, there were increases in the relative abundances of *Lactobacillus* and *Turicibacter*. Previous studies have demonstrated that *Lactobacillus* can mitigate galactose-induced liver injury in rats by suppressing hepatic inflammation, enhancing intestinal barrier function, and modulating the regulatory metabolome of gut microbiota [44]. *Turicibacter* belongs to the order *Bifidobacteria*, which regulates glycolipid metabolism as an intestinal probiotic. Additionally, it showed significant variations in several strains, including *Escherichia-Shigella*, *Uncultured bacterium Prevotellaceae NK3B31 group*, and *Uncultured bacterium Prevotellaceae UCG-001*. These strains were positively correlated with the control group (without treatment) and negatively correlated with the healthy control group and the MLNP (H)-treated group (Fig. 7g). As depicted in Fig. 7h, the *Firmicutes* level decreased, while the levels of *Bacteroidota* and *Patescibacteria* increased in the DEN/NMOR control group. In the gut, *Firmicutes* and *Bacteroidota* are dominant strains for gram-positive and gram-negative bacteria, respectively. Changes in their ratios indicate intestinal dysbiosis, which has been observed in various liver diseases [45]. Studies indicate that the majority of pro-inflammatory bacteria are classified as *Patescibacteria*, while numerous probiotics fall under *Firmicutes*. Intestinal disorders promote the proliferation of pro-inflammatory factor-producing bacteria and alter bile acid metabolism, thereby contributing to the development of HCC [46]. The MLNP (H)-treated group was able to reverse the changes mentioned above.

Studies have demonstrated that *Escherichia-Shigella*, a member of the *Enterobacteriaceae* family, exerts its pathogenicity in nonalcoholic fatty liver disease patients by modulating lipid metabolism and promoting lipid accumulation, ultimately resulting in varying degrees of hepatic steatosis, inflammation, and fibrosis [47]. The proportions of both *Prevotellaceae* and *Bacteroides* increased in patients with advanced liver cirrhosis [48]. Furthermore, there was no overlap in colony structure between the healthy mice and the DEN/NMOR control mice, with the latter being dispersed far apart. However, after oral administration of MLNPs to liver cancer mice, their colony structures of the MLNP (H)-treated group partially intersected with those of the healthy control mice (Fig. 7i). Representative cultures were chosen to showcase the trajectory of beneficial and detrimental bacteria following MLNP treatment (Fig. 7j-l). These results collectively imply that the treatment of MLNPs can efficiently modulate the balance of intestinal flora.

## Conclusion

Natural exosome-like nanovesicles were extracted and purified from fresh leaves of *Morus nigra* L. for the treatment of hepatocellular carcinoma. These *Morus nigra* L.-derived lipid nanoparticles (MLNPs) encompass a diverse array of functional constituents, comprising lipids, proteins, and flavonoids, with galactose identified as a potential target for liver tumor-targeting. Furthermore, they exhibited exceptional stability under simulated gastrointestinal conditions and demonstrated outstanding biocompatibility, thus making them highly suitable for in vivo application. The in vitro experiments revealed that galactose groups on the surface of MLNPs facilitated their specific internalization by Hepa1-6 cells and augmented their cytotoxicity against liver tumor cells. It was found that MLNPs caused cell cycle arrest at the G0/G1 phase and induced apoptosis in Hepa1-6 cells. They also triggered a surge in intracellular ROS levels and significantly inhibited the proliferation and migration of hepatoma cells. Oral administration of MLNPs showed superior biosafety compared with intravenous administration, without causing immunogenic or toxic side effects. In a murine model of primary hepatic carcinoma, oral MLNPs exhibited remarkable liver-targeting and enrichment capabilities, significantly suppressing tumor growth and modulating intestinal microbial balance. In summary, MLNPs represent a natural, safe, and eco-friendly nanomedicine with exceptional liver tumor-targeting capabilities, which can be exploited for oral treatment of hepatocellular carcinoma.

### Electronic supplementary material

Below is the link to the electronic supplementary material.

**Supplementary Material 1: Figure S1.** Percentages and MFIs of DiO-labeled MLNPs internalized by L929 cells after co-incubation for 1, 3, and 5 h, respectively. Each point represents the mean ± S.E.M. (n = 3; *p < 0.05, **p < 0.01, and ***p < 0.001). **Figure S2.** Hemolysis test. (a) Digital photo and (b) hemolysis rates of erythrocytes with the treatment of Triton X-100 (positive control; 0.1%, w/v), PBS (negative control), and MLNPs (various protein concentrations). Each point represents the mean ± S.E.M. (n = 3). **Figure S3.** (a) Body weight and (b) organ index of different mouse groups. The amounts of (c) ALT, (d) AST, (e) TBIL, (f) GGT, (g) CRE, and (h) BUN in the serum from different mouse groups. Each point represents the mean ± S.E.M. (n = 3; *p < 0.05). **Figure S4.** Blood test results from mice with different treatments. Each point represents the mean ± S.E.M. (n = 3; *p < 0.05). **Figure S5.** Histological analysis of the five principle organs (heart, liver, spleen, lung, and kidney) from the mice with the treatment of MLNPs *via i.v.* and oral routes (Scale bar = 200 µm, n = 3). **Figure S6.** (a) The organ index and (b) histological analysis of the four principle organs (heart, spleen, lung, and kidney) from mice with different treatments (Scale bar = 200 µm). Each point represents the mean ± S.E.M. (n = 5)

## Abbreviations

4T1: Mouse breast cancer cell line
A549: A549 human lung carcinoma cell line
AFM: Atomic force microscopy
ALT: Alanine aminotransferase
ASGPR: Asialoglycoprotein receptor
AST: Aspartate aminotransferase
BUN: Urea nitrogen
Cer: Ceramides
CRE: Creatinine
CT-26: Mouse colon carcinoma cell line
DAPI: 2-(4-amidinophenyl)-6-indolecar-bamidine dihydrochloride
DEN: Diethylnitrosamine
DiO: 3,3ʹ-dioctadecyloxacarbocyanine perchlorate
DiR: 1,1ʹ-dioctadecyl-3,3,3ʹ,3ʹ-tetramethylindotricarbocyanine iodide
DLS: Dynamic light scattering
DMEM: Dulbecco’s modified eagle medium
DMSO: Dimethyl sulfoxide
GIT: Gastrointestinal tract
GO: Gene ontology
H&E: Hematoxylin and eosin
HCC: Hepatocellular carcinoma
Hepa1-6: Hepa1-6 murine hepatoma cell line
HepG2: Hepatoma G2 cell line
HexCer: Hexose ceramides
IC_50_: Half maximal inhibitory concentrations
*i.g.*: Oral gavage
*i.v.*: Intravenous
K3G: Kaempferol-3-*O*-glucoside
KEGG: Kyoto encyclopedia of genes and genomes
L929: L929 murine fibroblast cell line
LPC: Hemolytic phosphatidylcholine
MC3T3-E1: MC3T3-E1 mouse calvaria-derived pre-osteoblast cell line
MGDG: Monogalactodiglycerol
MLNPs: Mulberry leaf lipid nanoparticles
MTT: Methylthiazolyldiphenyl-tetrazolium bromide
NMOR: *N*-nitrosomorpholine
PBS: Phosphate buffered saline
PCoA: Principal coordinates analysis
PDI: Polydispersity index
PI: Propidium iodide
Q3G: 3-*O-*glucoside
ROS: Reactive oxygen species
TBA: Total bile acids
TEM: Transmission electron microscopy
TG: Triglycerides
TUNEL: Terminal deoxynucleotidyl transferase-mediated dUTP-biotin nick end labeling
