# Supplementary material for: Natural lipid nanoparticles extracted from Morus nigra L. leaves for targeted treatment of hepatocellular carcinoma via the oral route
Source: J Nanobiotechnology. 2024 Jan 3;22:4. doi: 10.1186/s12951-023-02286-3 (PMC10763359; doi:10.1186/s12951-023-02286-3)
Supplement: Supplementary file 1 — Supplementary Material 1: Figure S1. Percentages and MFIs of DiO-labeled MLNPs internalized by L929 cells after co-incubation for 1, 3, and 5 h, respectively. Each point represents the mean ± S.E.M. (n = 3; *p < 0.05, **p < 0.01, and ***p < 0.001). Figure S2. Hemolysis test. (a) Digital photo and (b) hemolysis rates of erythrocytes with the treatment of Triton X-100 (positive control; 0.1%, w/v), PBS (negative control), and MLNPs (various protein concentrations). Each point represents the mean ± S.E.M. (n = 3). Figure S3. (a) Body weight and (b) organ index of different mouse groups. The amounts of (c) ALT, (d) AST, (e) TBIL, (f) GGT, (g) CRE, and (h) BUN in the serum from different mouse groups. Each point represents the mean ± S.E.M. (n = 3; *p < 0.05). Figure S4. Blood test results from mice with different treatments. Each point represents the mean ± S.E.M. (n = 3; *p < 0.05). Figure S5. Histological analysis of the five principle organs (heart, liver, spleen, lung, and kidney) from the mice with the treatment of MLNPs via i.v. and oral routes (Scale bar = 200 µm, n = 3). Figure S6. (a) The organ index and (b) histological analysis of the four principle organs (heart, spleen, lung, and kidney) from mice with different treatments (Scale bar = 200 µm). Each point represents the mean ± S.E.M. (n = 5) [file 12951_2023_2286_MOESM1_ESM.docx]

**Additional file 1**

**Natural lipid nanoparticles extracted from *Morus nigra* L. leaves for targeted treatment of hepatocellular carcinoma *via* the oral route**

Qiang Gao^a,†^, Nanxi Chen ^a,†^, Baoyi Li^a,†^, Menghang Zu^a^, Ya Ma^a^, Haiting Xu^a,b^, Zhenhua Zhu^c^, Rui L. Reis^d,e^, Subhas C. Kundu^d,e^, Bo Xiao^a,*^

^a^State Key Laboratory of Resource Insects, College of Sericulture, Textile, and Biomass Sciences, Southwest University, Beibei, Chongqing 400715, China

^b^State Key Laboratory of Southwestern Chinese Medicine Resources, Pharmacy School, Chengdu University of Traditional Chinese Medicine, Chengdu, Sichuan 611137, China

^c^Department of Gastroenterology, The First Affiliated Hospital of Nanchang University, Nanchang, Jiangxi 330006, China

^d^3Bs Research Group, I3Bs — Research Institute on Biomaterials, Biodegradables and Biomimetics, University of Minho, Headquarters of the European Institute of Excellence on Tissue Engineering and Regenerative Medicine, AvePark, Barco 4805-017, Guimaraes, Portugal

^e^ICVS/3B’s-PT Government Associate Laboratory, Braga, Guimarães, Portugal

∗ Corresponding author

E-mail address: bxiao@swu.edu.cn (B. Xiao)

† These authors contributed equally to this work.

**Supplementary experimental details**

**Physicochemical property of MLNPs**

Physicochemical properties of MLNPs were detected. 10 µL of MLNPs were dispersed in 1 mL of secondary water, and the hydrodynamic particle size (nm), size distribution, and zeta potential (mV) of MLNPs were characterized by using DLS instrument (Malvern, Nano ZS90, Worcestershire, UK).

Lipidomic analysis of total lipids from MLNPs was performed by Shanghai Minxin Biotechnology Co., Ltd. (Shanghai, China). In brief, the lipid composition of MLNPs was determined by using a triple quadrupole mass spectrometer (an Applied Biosystems Q-TRAP, Applied Biosystems, Foster City, CA). The data are reported as the percentage of the total signals for the molecular species determined after normalization of the signals to internal standards of the same lipid classes.

Proteins from MLNPs were analyzed by Majorbio Bio-pharm Technology Co.,Ltd. (Shanghai, China). In brief, the lysis buffer ( 8 M urea, 1 % SDS, containing protease inhibitor ) was added according to the sample / lysis buffer volume ratio of 1 : 10. After ultrasonication on ice for 2 min, it was placed for 30 min to fully lyse. After that, it was centrifuged at 12,000 × g for 30 min to collect the supernatant. Samples proteins were identified and quantified by LC-MS/MS (liquid chromatography coupled with tandem mass spectrometry) using Orbitrap mass spectrometry (Thermo Fisher Scientific, Bremen, Germany). Finally, the data were analyzed based on the National Center for Biotechnology Information (NCBI) database.

**Stability detection of MLNPs**

To assess the stability of MLNPs in different sections of the gastrointestinal tract (GIT) and blood, they were suspended in simulated gastric fluid (pH = 1.2), simulated small intestinal fluid (pH = 6.8), and simulated colonic fluid (pH = 7.4) respectively. Following incubation for different time intervals (8, 24, and 72 h) at a temperature of 37 °C, the hydrodynamic particle size (nm) and zeta potential (mV) of MLNPs were determined using DLS measurements.

**Cell culture**

HepG2, Hepa1-6, 4T1, CT26.WT, A549, and L929 cell lines were purchased from the Center for Excellence in Molecular Cell science, Chinese Academy of Sciences (Shanghai, China). And the MC3T3-E1 cell line was gifted by the Rao Xi research group of the college of Materials and Energy, Southwest University. The cells were cultured in Dulbecco's Modified Eagle's medium (DMEM) supplemented with 10% fetal bovine serum and 1% penicillin/streptomycin and maintained in a CO2 incubator at 37 °C.

**Live/dead assay**

Hepa1-6 cells were inoculated into a 12-well plate with a density of 1.5 × 10^5^ cells per well and incubated overnight. Then, the cells were treated with 50 μg/mL MLNPs for 6, 12, and 24 h, respectively. After co-incubation, the cells were washed 3 times with cold PBS. Subsequently, 500 µL of calcein-AM and propidium iodide (PI) were added to each well and incubated at 37 ℃ for 30 min in a dark environment. Afterward, the dye was removed and cells were washed 3 times with PBS. Finally, the samples were imaged immediately using a fluorescent microscope (Olympus, IX73, Tokyo, Japan).

***In vitro* pro‑apoptosis property of MLNPs**

Hepa1-6 cells were seeded in a 12-well plate at a density of 2 × 10^5^ cells per well and incubated overnight. Then, the cells were treated with 50 μg/mL MLNPs for 6, 12, and 24 h, respectively. After co-incubation, the cells were washed 3 times with cold PBS and digested with trypsin (300 µL per well) before being centrifuged at 1000 × g for 3 min. The supernatant was discarded, and 195 µL of probe binding solution was added to each tube for resuspending the cell precipitation. Subsequently, 5 µL of Annexin FITC and 10 µL of PI were sequentially introduced into the sample. The mixture was then incubated at a temperature of 25 ℃ in a dark environment for a duration of 20 min. Finally, the cells were analyzed using flow cytometry (FCM, Beckman Coulter Inc, USA).

***In vitro* wound-healing properties of MLNPs**

The wound-healing properties of MLNPs were evaluated using a scratch assay. Hepa1-6 cells were inoculated into a 6-well plate with a density of 5 × 10^5^ cells per well and incubated overnight. Scratches were created in each well using a pipet tip, followed by washing with PBS. Subsequently, the 'wounds' were imaged both before and after the addition of MLNPs suspensions (50 μg/mL). The wound-healing capabilities of MLNPs were assessed by measuring the remaining unhealed area utilizing ImageJ software.

**Transwell invasion assay**

Cell invasion assay was performed based on a Transwell plate (pore size: 8 mm; Nest Biotechnology, Wuxi, China). Hepa1-6 cells were seeded into the upper chamber at a density of 1 × 10^4^ per well and incubated overnight. The upper chambers were filled with serum-free media containing MLNPs suspensions (50 μg/mL), while the lower chamber was supplemented with the media containing FBS (10%, v/v) as a chemoattractant to induce cell migration across the membrane. After 24 hours of incubation, cells on the upper membrane surface were removed with humid cotton buds, while those on the bottom surface were fixed with paraformaldehyde (1%, v/v) and stained with crystal violet (0.1%, w/v) for 20 min. The number of cells in five microscopic fields per well was counted to determine cell migration in the lower part of the inserts, and results were expressed as an average number of cells per field.

***In vitro* cellular uptake profiles of MLNPs**

DiO-labeled MLNPs were utilized to quantitatively and qualitatively investigate the cellular uptake profiles of MLNPs. Briefly, 2 μL of DiO solution (10 mg/mL) was added to MLNPs suspensions (protein concentration: 50 μg/mL, 1 mL). After incubation in darkness at 37 ℃ for 30 min, unbound DiO was removed by centrifugation at 150,000 × g for 1 h at 4 ℃, and the purified DiO-labeled MLNPs were obtained.
 Hepa1-6 and L929 cells were seeded in 12-well plates at a density of 2 × 10^5^ per well and incubated overnight. Thereafter, the complete culture medium was replaced with serum-free medium containing DiO-labeled MLNPs (protein concentration: 50 μg/mL). After co-incubation for different time intervals (1, 3, and 5 h), the excess NPs were eliminated by washing the cells 3 times with PBS. Subsequently, the cells were collected, re-suspended in PBS, and analyzed using FCM (Beckman Coulter Inc., USA) based on 10,000 gated cell events.

Hepa1-6 and L929 cells were seeded in 12-well plates at a density of 2 × 10^5^ per well and incubated overnight. Then, the complete culture medium was replaced with serum-free medium containing DiO-labeled MLNPs (protein concentration: 50 μg/mL). After co-incubation for different time intervals (1, 3, and 5 h), the cells were washed with PBS 3 times to remove excess NPs and then fixed with a solution of paraformaldehyde (4%, v/v) for 20 min. Thereafter, rhodamine phalloidin and DAPI were used to stain cytoskeleton and nucleus, respectively. Finally, the fluorescent images of cells were acquired using a high-resolution laser confocal microscope (CLSM, Olympus, FV-3000, Japan).

**Impacts of MLNPs on mitochondrial membrane potential**

Hepa1-6 and L929 cells were seeded at a density of 2 × 10^5^ cells per well in 12-well plates and incubated overnight. Then, the cells were co-cultured with MLNPs at a concentration of 50 µg/mL for 12, 24, and 48 h, respectively. After co-incubation, cells were washed with cold PBS 3 times. Hoechst 33342 (1ml) was then added to each well and incubated at 37 ℃ in a dark environment for 30 min. Subsequently, JC-1 staining solution (1 mL) was added to each well and incubated at 37 ℃ in a dark environment for another 30 min. The staining solution was aspirated, and 1 mL of DMEM culture solution was added to each well. Subsequently, the samples were imaged using a CLSM (Olympus, FV-3000, Japan).

**Impacts of MLNPs on intracellular ROS generation**

Hepa1-6 and L929 cells were seeded in a 12-well plate at a density of 2 × 10^5^ cells per well and incubated overnight, respectively. Subsequently, the cells were co-cultured with MLNPs at a concentration of 50 μg/mL for 12 and 24 h, respectively. After co-incubation, cells were washed with cold PBS and stained with Hoechst 33342 and DCFH-DA for 30 minutes each at 37 ℃ in the dark. The staining solution was removed and replaced with DMEM culture solution before imaging using a CLSM (Olympus, FV-3000, Japan).

**Impacts of MLNPs on cell cycle**

Hepa1-6 cells were inoculated into a 12-well plate with a density of 2 × 10^5^ cells per well and incubated overnight. Cells were co-cultured with 50 μg/mL MLNPs for 12 and 24 h, respectively. And then the cells were digested by trypsin, fixed in 70% ethanol (4 °C) for 12 h, and treated with PI and RNase A for 30 min after being collected by centrifugation and washed with cold PBS 3 times. Finally, cells were analyzed by FCM (Beckman Coulter Inc, USA).

***In vivo* targeting verification of MLNPs**

6-Week-old female C57BL/6J mice were obtained from Chongqing Byrness Weil Biotechnology Co. Ltd. (Chongqing, China). Mice investigations were approved by the Institutional Animal Care and Use Committee of Southwest University. 150 μL of 10% chloral hydrate solution was injected intraperitoneally per mouse. After complete anesthesia, the skin and peritoneum layers beneath the sword cartilage were sequentially incised with an approximately 1 cm opening. Gentle compression on both sides of the ribs facilitated liver protrusion. 50 μL of Hepa1-6 cells (5 × 10^7^ cells/mL) were injected into the liver at a slight tilt. After one week, three mice were randomly dissected to observe liver tumor formation. To track the in vivo bio-distribution of MLNPs, DiR was used for labeling. Mice were orally administered DiR-MLNPs at a concentration of 5 mg protein/kg per mouse. Mice were euthanized at predetermined time points (12, 24, 48, and 72 h). Subsequently, the major organs and GITs were isolated and imaged for fluorescence using an IVIS spectrum imaging system (PerkinElmer/Caliper LifeSciences, Hopkinton, MA, USA).

**Establishment of a model for primary liver carcinoma**

6-Week-old female ICR mice were obtained from Chongqing Byrness Weil Biotechnology Co. Ltd. (Chongqing, China). Mice investigations were approved by the Institutional Animal Care and Use Committee of Southwest University. Except for the healthy group, each mouse was intraperitoneally injected with 20 mg/kg DEN (10 μL/g) and their drinking water was replaced with 80 ppm NMOR. After continuous consumption for 24 weeks, the dosing group received oral administration of MLNPs at a dose of either 2.5 mg/kg or 5 mg/kg every three days, totaling five doses administered. Mice were monitored for changes in their status, body weight, and survival rate throughout the duration of the experiment. On the 16th day, the mice were dissected and their major organs, blood, and feces were collected for analysis.

***In vitro* hemolysis assay**

Briefly, fresh blood was collected from the eyelid posterior sinus vein of FVB mice and centrifuged at 3000 × g for 5 min, washed 3 times, and suspended in PBS (2%, v/v). Meanwhile, MLNPs with different concentrations (1, 2, 5, 10, 20, 50, and 100 μg/mL) were co-cultured with erythrocytes solutions for 1 h at 37 ℃ before being centrifuged at 3000 × g for 5 min. Finally, hemoglobin amounts in the supernatants were analyzed by spectrophotometric tests at 570 nm. The untreated erythrocyte suspension was utilized as a negative control, whereas erythrocyte suspension treated with Triton X-100 (1%, w/v) was used as a positive control.

**
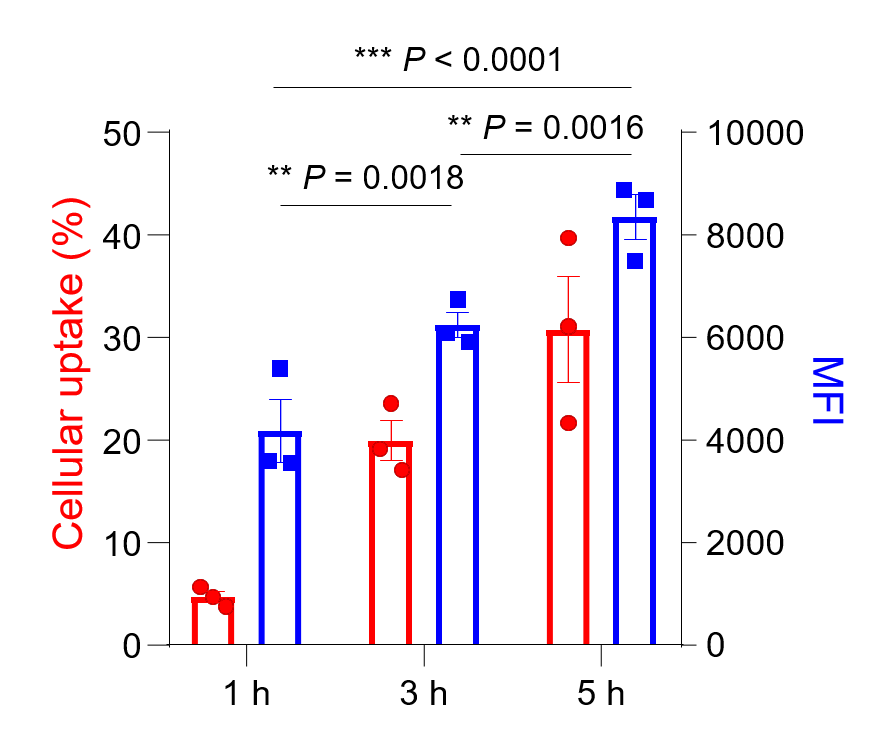
**

**Figure S1.** Percentages and MFIs of DiO-labeled MLNPs internalized by L929 cells after co-incubation for 1, 3, and 5 h, respectively. Each point represents the mean ± S.E.M. (n = 3; **p* < 0.05, ***p* < 0.01, and ****p* < 0.001).


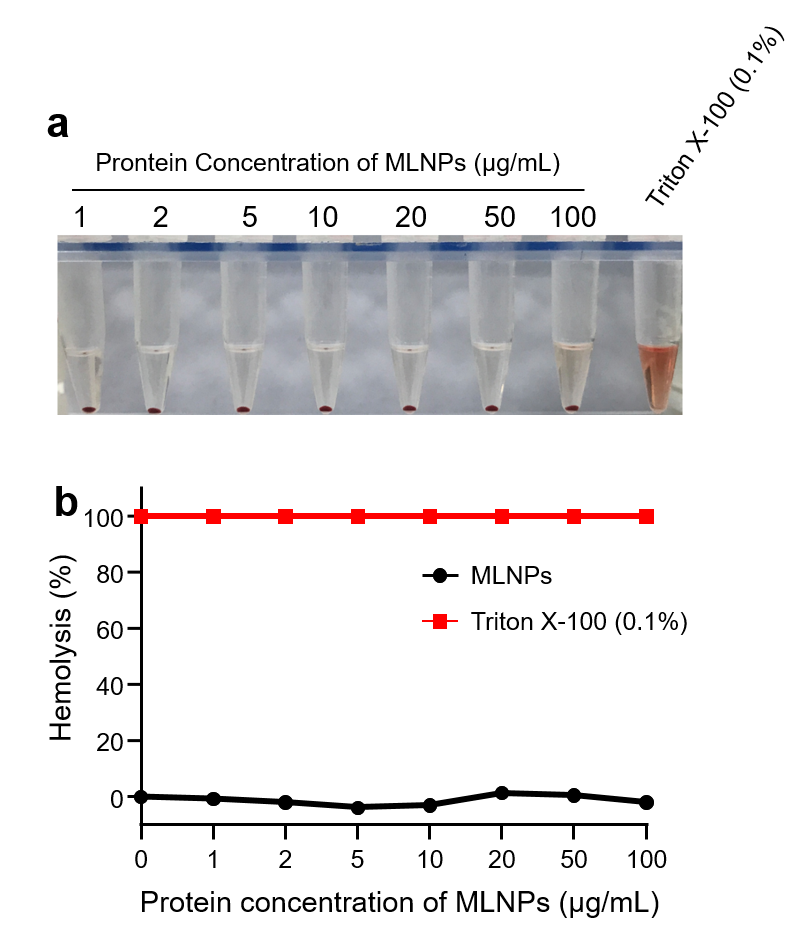


**Figure S2.** Hemolysis test. (a) Digital photo and (b) hemolysis rates of erythrocytes with the treatment of Triton X-100 (positive control; 0.1%, w/v), PBS (negative control), and MLNPs (various protein concentrations). Each point represents the mean ± S.E.M. (n = 3).

**
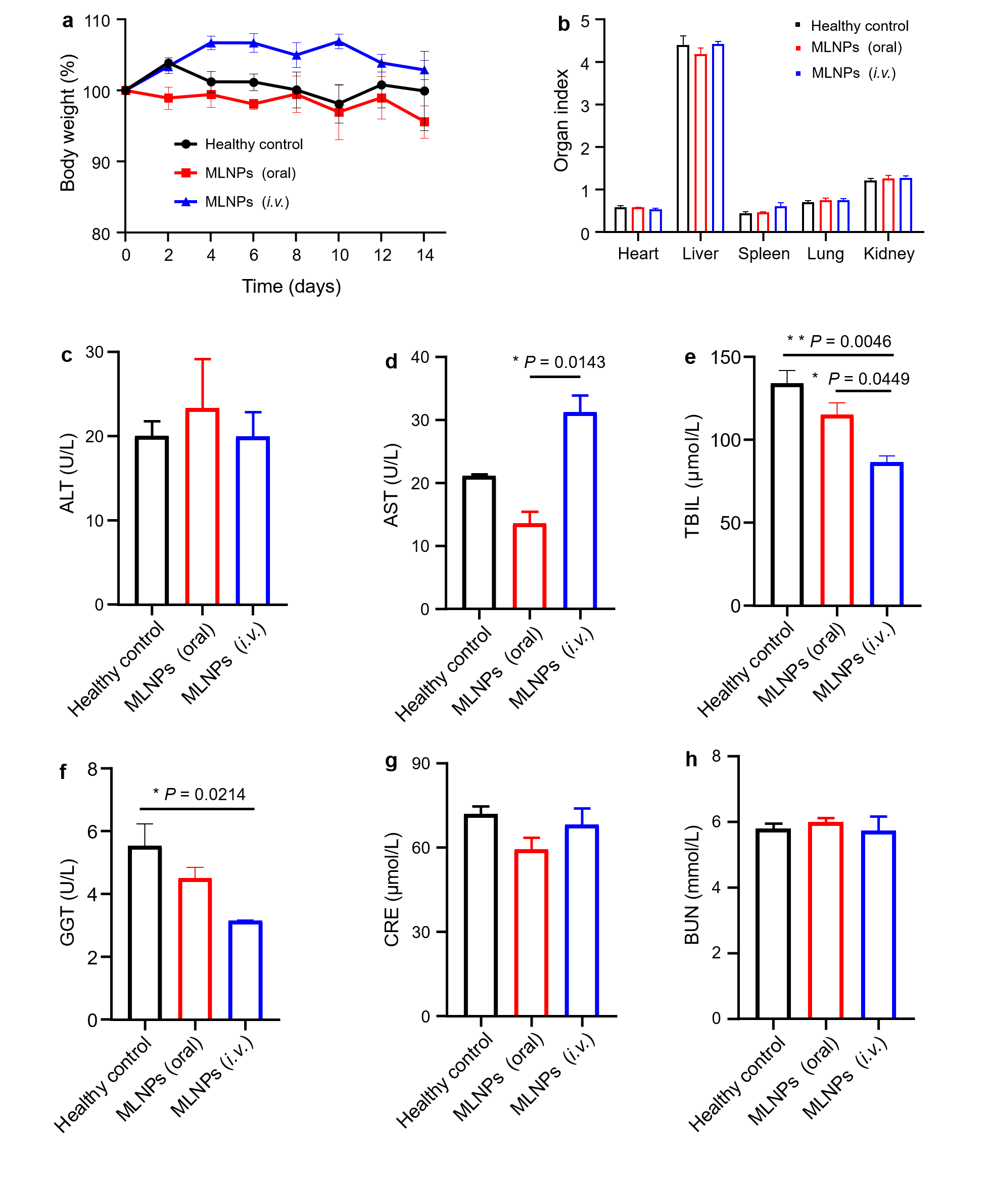
**

**Figure S3.** (a) Body weight and (b) organ index of different mouse groups. The amounts of (c) ALT, (d) AST, (e) TBIL, (f) GGT, (g) CRE, and (h) BUN in the serum from different mouse groups. Each point represents the mean ± S.E.M. (n = 3; **p* < 0.05).

**
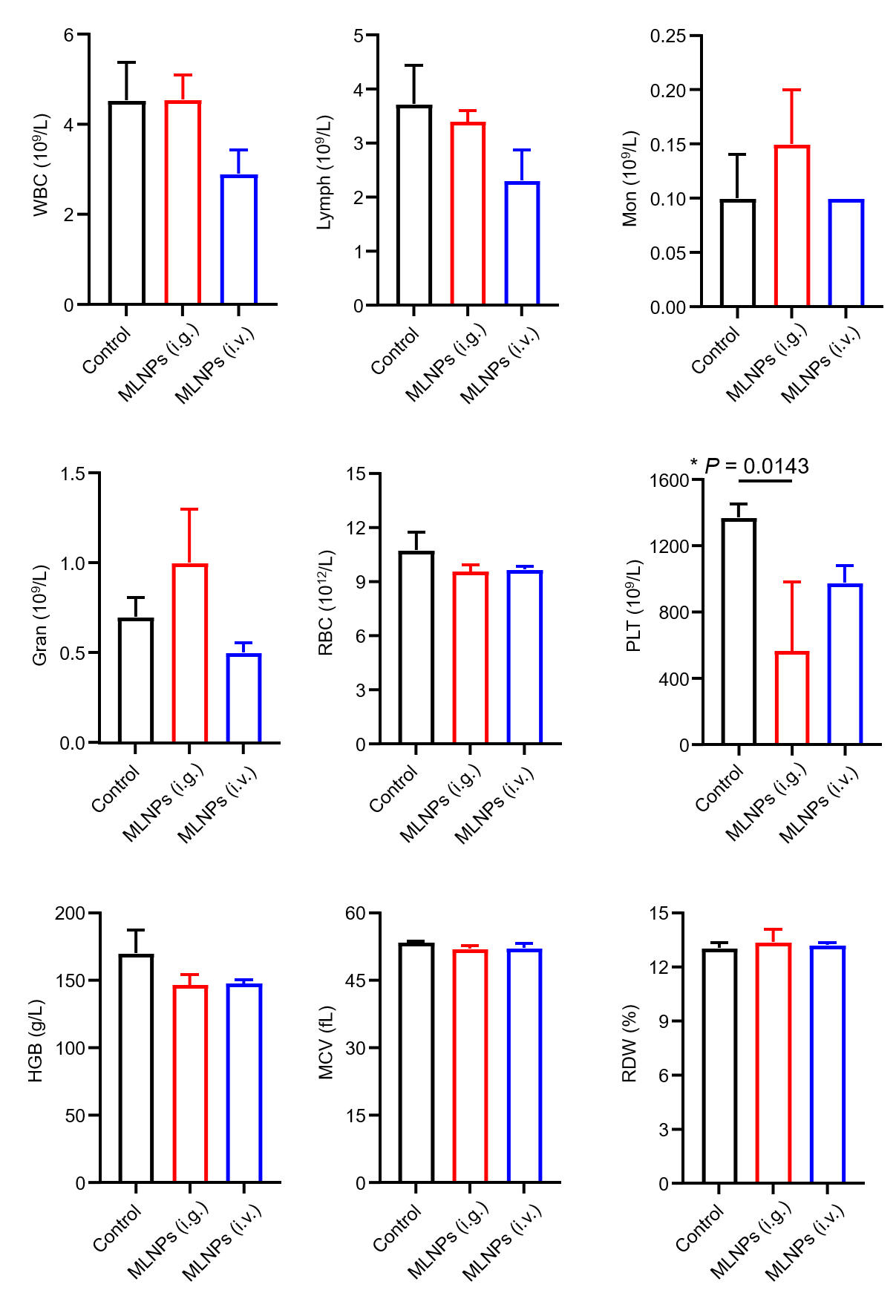
**

**Figure S4.** Blood test results from mice with different treatments. Each point represents the mean ± S.E.M. (n = 3; **p* < 0.05).


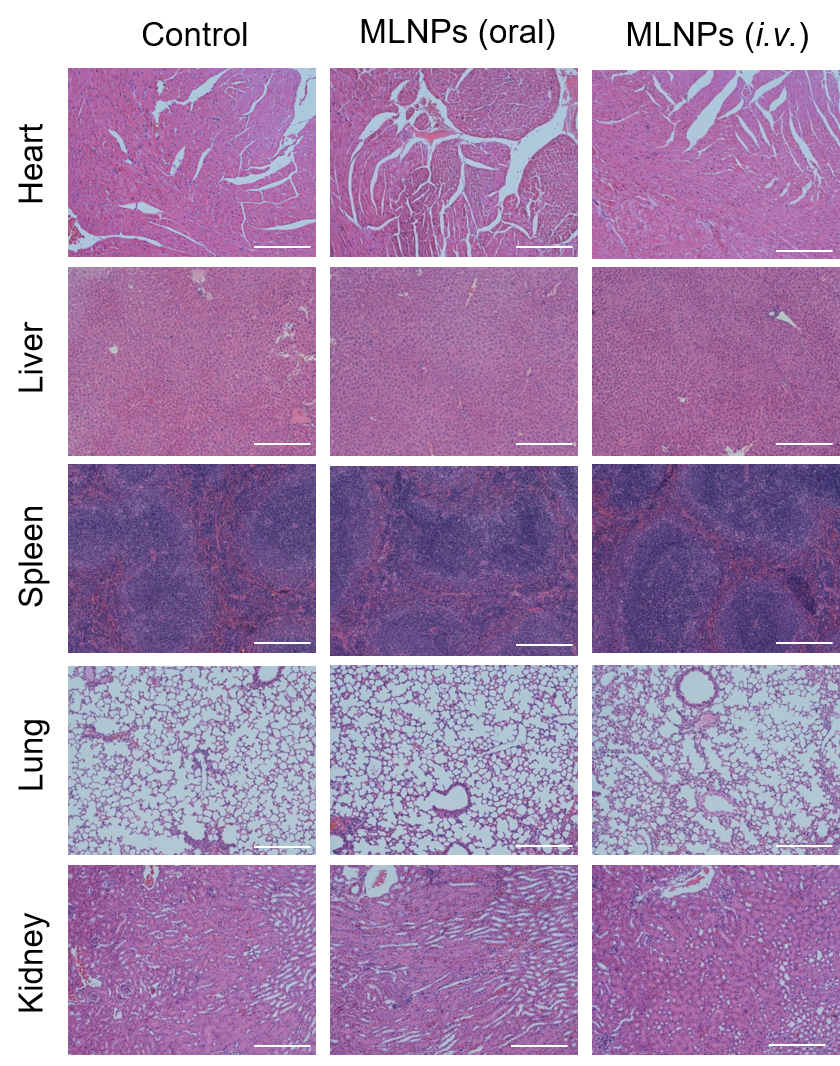


**Figure S5**. Histological analysis of the five principle organs (heart, liver, spleen, lung, and kidney) from the mice with the treatment of MLNPs *via* *i.v.* and oral routes (Scale bar = 200 μm, n = 3).

**
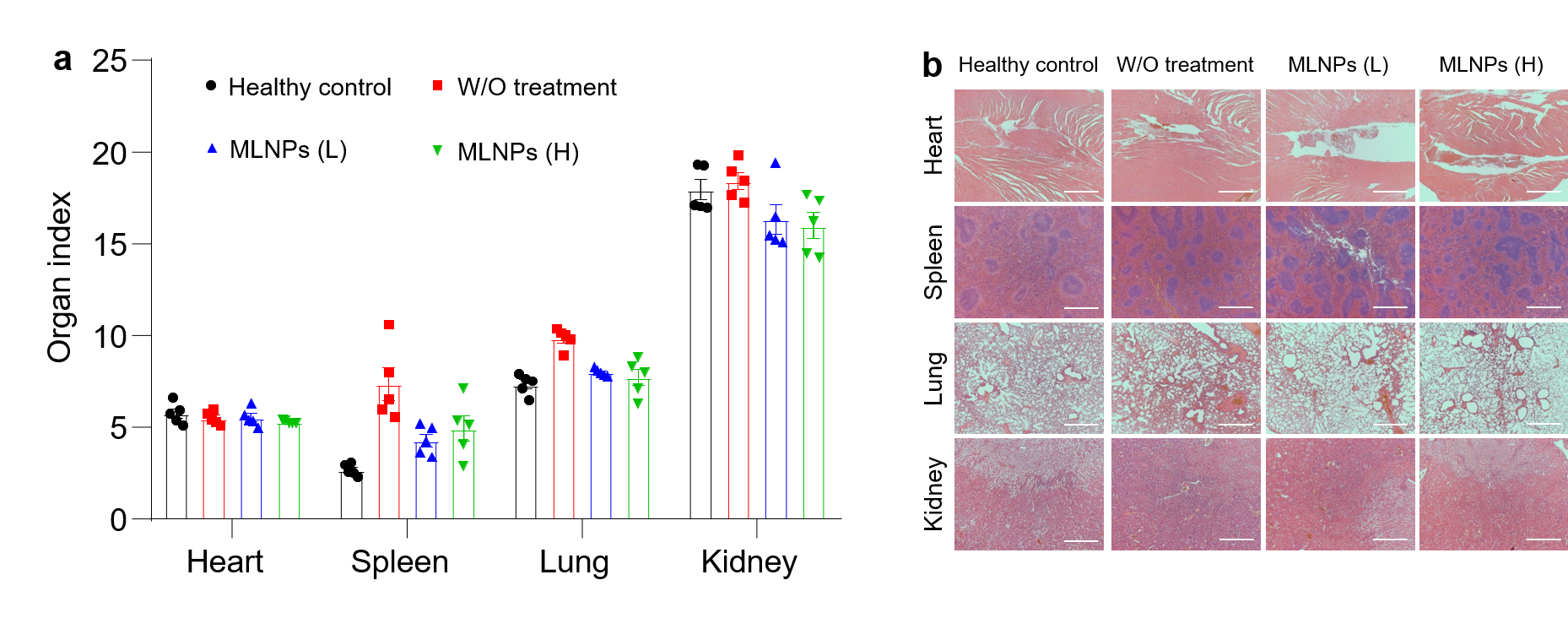
Figure S6**. (a) The organ index and (b) histological analysis of the four principle organs (heart, spleen, lung, and kidney) from mice with different treatments (Scale bar = 200 μm). Each point represents the mean ± S.E.M. (n = 5).
